# The Effects of Knockdown of Rho-Associated Kinase 1 and Zipper-Interacting Protein Kinase on Gene Expression and Function in Cultured Human Arterial Smooth Muscle Cells

**DOI:** 10.1371/journal.pone.0116969

**Published:** 2015-02-27

**Authors:** Jing-Ti Deng, Xiu-Ling Wang, Yong-Xiang Chen, Edward R. O’Brien, Yu Gui, Michael P. Walsh

**Affiliations:** 1 Smooth Muscle Research Group and Department of Biochemistry and Molecular Biology, University of Calgary, Alberta, Canada; 2 Southern Alberta Cancer Research Institute Microarray and Genomics Facility, University of Calgary, Alberta, Canada; 3 Division of Cardiology, Libin Cardiovascular Institute of Alberta, University of Calgary, Alberta, Canada; 4 Department of Physiology and Pharmacology, University of Calgary, Alberta, Canada; 5 Hotchkiss Brain Institute and Libin Cardiovascular Institute of Alberta, University of Calgary, Alberta, Canada; Hungarian Academy of Sciences, HUNGARY

## Abstract

Rho-associated kinase (ROCK) and zipper-interacting protein kinase (ZIPK) have been implicated in diverse physiological functions. ROCK1 phosphorylates and activates ZIPK suggesting that at least some of these physiological functions may require both enzymes. To test the hypothesis that sequential activation of ROCK1 and ZIPK is commonly involved in regulatory pathways, we utilized siRNA to knock down ROCK1 and ZIPK in cultured human arterial smooth muscle cells (SMC). Microarray analysis using a whole-transcript expression chip identified changes in gene expression induced by ROCK1 and ZIPK knockdown. ROCK1 knockdown affected the expression of 553 genes, while ZIPK knockdown affected the expression of 390 genes. A high incidence of regulation of transcription regulator genes was observed in both knockdowns. Other affected groups included transporters, kinases, peptidases, transmembrane and G protein-coupled receptors, growth factors, phosphatases and ion channels. Only 76 differentially expressed genes were common to ROCK1 and ZIPK knockdown. Ingenuity Pathway Analysis identified five pathways shared between the two knockdowns. We focused on cytokine signaling pathways since ROCK1 knockdown up-regulated 5 and down-regulated 4 cytokine genes, in contrast to ZIPK knockdown, which affected the expression of only two cytokine genes (both down-regulated). IL-6 gene expression and secretion of IL-6 protein were up-regulated by ROCK1 knockdown, whereas ZIPK knockdown reduced IL-6 mRNA expression and IL-6 protein secretion and increased ROCK1 protein expression, suggesting that ROCK1 may inhibit IL-6 secretion. IL-1β mRNA and protein levels were increased in response to ROCK1 knockdown. Differences in the effects of ROCK1 and ZIPK knockdown on cell cycle regulatory genes suggested that ROCK1 and ZIPK regulate the cell cycle by different mechanisms. ROCK1, but not ZIPK knockdown reduced the viability and inhibited proliferation of vascular SMC. We conclude that ROCK1 and ZIPK have diverse, but predominantly distinct regulatory functions in vascular SMC and that ROCK1-mediated activation of ZIPK is not involved in most of these functions.

## Introduction

Rho-associated kinase (ROCK) and zipper-interacting protein kinase (ZIPK) are serine/threonine protein kinases that have been implicated in a variety of important physiological functions, including smooth muscle contraction, cell proliferation, cell adhesion, apoptosis, cell migration and inflammation [1–6].

ROCK belongs to a kinase family that is primarily activated by interaction with the small GTPase RhoA [7–9]. Two isoforms, ROCK1 and ROCK2, have been identified, which share > 90% sequence identity in the *N*-terminal kinase domain, while the *C*-terminal regulatory domains show significant divergence [3,10]. The widespread tissue distributions of ROCK1 and 2 are similar, and relatively few studies have identified isoform-specific roles of ROCK. Several substrates of ROCK have been identified, including MYPT1 (the myosin targeting subunit of myosin light chain phosphatase), ERM (ezrin-radixin-moesin) family members, adducin, vimentin and LIM (Lin11, Isl1 and Mec3)-kinase [3].

ZIPK is a member of the family of death-associated protein kinases (DAPKs) [1,6,11] with five members: DAPK1, DAPK2, DAPK3 (ZIPK), DRAK1 and DRAK2 (DAPK-related apoptosis-inducing protein kinases 1 and 2). The catalytic domains of DAPKs1–3 exhibit ~ 80% sequence identity, whereas this region of DRAKs1 and 2 exhibits ~ 50% identity [12]. ZIPK contains an *N*-terminal kinase domain, followed by a putative autoinhibitory domain and a *C*-terminal leucine zipper domain, which is thought to mediate homodimerization and is required for the death-promoting effects of the kinase. In contrast to the other DAPKs, ZIPK does not contain a calmodulin-binding domain, consistent with the fact that its activity is not regulated by Ca^2+^ [13]. It also lacks a death domain found in other family members, but does contain three nuclear localization signals. DAPKs1–3 exhibit widespread tissue distribution in mice and rats [14]. ZIPK has been implicated in cell death (apoptosis and autophagy) [13], transcriptional regulation, inflammatory signaling [15], cell motility [2,4] and smooth muscle contraction [16]. Several substrates of ZIPK have been identified, including STAT3 (signal transducer and activator of transcription 3) [17], myosin II regulatory light chains (LC_20_) [18,19], CPI-17 (the 17-kDa protein kinase C-potentiated inhibitory protein of type 1 protein serine/threonine phosphatase) [20] and MYPT1 [18,21]. ZIPK activity is regulated by phosphorylation [22]. Phosphorylation of three sites within the kinase domain (Thr180, Thr225 and Thr265) is required for kinase activation [22]. DAPK1 [23] and ROCK1 [24] have been implicated as ZIPK kinases and Thr265 has been identified as a ZIPK autophosphorylation site whose phosphorylation is required for full catalytic activity and cell death [25]. ZIPK expression is increased in the aorta and mesenteric artery of spontaneously hypertensive compared to normotensive Wistar Kyoto rats [26] and ZIPK has been shown to mediate reactive oxygen species (ROS)-dependent vascular inflammation, potentially leading to hypercontractility and hypertrophy associated with hypertension [27].

Both ROCK and ZIPK have been implicated in the phosphorylation of smooth muscle and non-muscle LC_20_ and, therefore, regulation of smooth muscle and non-muscle contractility and motility [18,19,28–30]. Prevailing evidence, however, suggests that while LC_20_ is likely to be a physiological substrate of ROCK in non-muscle cells, this is not the case in smooth muscle cells [31–36]. In both smooth muscle and non-muscle cells and tissues, ZIPK was found to phosphorylate LC_20_ at both Ser19 and Thr18 in a Ca^2+^-independent manner [4,18,19]. Phosphorylation or activation of myosin is considered to contribute to the reorganization of actin filaments [37], cell motility [4] and cell death [38] in both smooth muscle and non-muscle cells. The impairment of these processes has been shown to be involved in several pathological conditions, such as hypertension, stroke, vasospasm, atherosclerosis, heart failure, pulmonary hypertension and various cancers [39–44].

Many aspects of regulation via ROCK and ZIPK pathways remain unclear. For example, while ROCK1 has been shown to phosphorylate and activate ZIPK [24], it is unclear if this linear relationship is common in the regulation of physiological processes by these two kinases. To test this hypothesis, we utilized a siRNA approach in combination with microarray analysis using a whole-transcript expression chip to identify changes in gene expression profiles induced by ROCK1 and ZIPK knockdown. Cytokine signaling and cell cycle regulatory pathways were particularly affected, which prompted functional assays to test the involvement of these kinases in cytokine production, cell viability and cell proliferation. To our knowledge, this is the first such gene expression study to be performed and leads to the identification of novel roles for these protein kinases and the conclusion that direct activation of ZIPK by ROCK1 is not a universal mechanism in signal transduction.

## Materials and Methods

### Materials

All chemicals were reagent grade unless indicated otherwise.

### Primary human arterial smooth muscle cell culture

Human coronary artery smooth muscle cells (CASMC: CC-2583) and human umbilical artery smooth muscle cells (UASMC: CC-2579) were purchased from Lonza and cultured in smooth muscle growth medium (Lonza SmGM-2: CC-3182) containing 5% fetal bovine serum (FBS), growth factors (hEGF, insulin and hFGF-β), gentamicin and amphotericin-B at 37°C in a humidified incubator with 5% CO_2_. These SMC maintain their morphological and phenotypic characteristics for up to 10 passages; therefore, cells were used for experiments within 10 passages.

### siRNA transfection

Human ROCK1 siRNA (purchased from Santa Cruz Biotechnology; sc-29473) consists of three target-specific siRNAs (19–25 nucleotides). Human ZIPK siRNA (purchased from Ambion; part number 4390824, siRNA ID #s559, Lot #ASO0UMMU), has the following sequence: sense 5′-GAGGAGUACUUCAGCAACAtt-3′, and antisense 5′-UGUUGCUGAAGUACUCCUCgt-3′. Negative control siRNA (Ambion negative control #2, part #AM4613) contains sequences that do not target any gene product. Cultured human CASMC and UASMC were transfected with siRNAs targeting ROCK1, ZIPK or negative control using HiPerFect Reagent (Qiagen) according to the manufacturer’s protocol. Briefly, on the day of transfection cells were seeded into a 4-well plate at a density of 6 x 10^4^ cells per well in 100 μL of complete SmGM and incubated under normal growth conditions. siRNAs were diluted in 100 μL of medium without serum to give a final concentration of 20 nM. After mixing by vortexing, HiPerFect Reagent (3 μL) was added to the diluted siRNA and mixed by vortexing for 10 s. The HiPerFect/siRNA complexes were added to the cells after incubation for 10 min at room temperature. The cells were incubated at 37°C in a CO_2_ incubator for another 4 h and a further 300 μL of complete culture medium were added. The transfected cells were incubated under normal growth conditions and the medium was changed at 30 h after transfection. Cells were cultured for another 18 h and both cells and culture supernatants were harvested at 48 h after transfection. Cells were used for RNA isolation or western blotting, and supernatants were used for ELISA to determine interleukin-6 (IL-6) and other cytokine levels (see below). To determine transfection efficiencies, CASMC and UASMC were co-transfected with FITC-labeled control siRNA and either ROCK1- or ZIPK-targeted or control siRNA, nuclei were labeled with DAPI and transfected cells were detected by fluorescence confocal microscopy. In each case, > 90% of the cells were fluorescently labeled.

### RNA isolation and cDNA microarrays

Total RNA was isolated from CASMC using the RNeasy Micro kit (Qiagen) according to the manufacturer’s instructions. The isolated RNA was quantified by UV absorption with a NanoDrop 1000 (NanoDrop Technologies, Inc.) and the quality verified with an Agilent RNA 6000 NanoChip on a 2100 Bioanalyzer (Agilent Technologies). Samples with an RNA integrity number (RIN) ≥ 8 were considered suitable for use in microarrays. A total of 250 ng of RNA in each sample with RIN > 9 was reverse transcribed, amplified, labeled with the Ambion WT Express kit (Ambion) and hybridized to Human Gene 1.0 ST arrays (Affymetrix) at 45°C for 16 h. The arrays were washed and stained on an Affymetrix GeneChip Fluidics-450 following the manufacturer’s protocol and scanned on an Affymetrix GeneChip Scanner 3000 7G System. The raw data sets for array comparisons have been deposited in the Gene Expression Omnibus website: www.ncbi.nlm.nih.gov/geo/ (accession number: GSE56819). Of the 28,868 genes represented on the GeneChip Human Gene 1.0ST array, genes for which the log 2-transformed signal intensities differed between the negative control and ROCK1- or ZIPK-knockdown groups by more than 0.585 (1.5-fold change without adjustment) were identified as genes whose expression was altered significantly by the kinase knockdown.

### Cell viability assay

Cell viability after kinase knockdown was evaluated with the MTT assay. 3-(4,5-Dimethyl-thiazol-2-yl)-2,5-diphen yltetrazolium bromide (MTT) was purchased from Sigma-Aldrich. After transfection with the siRNA/HiPerFect complexes for 30 h, the medium was replaced with fresh complete medium and the cells were cultured for a further 18 h. An amount of MTT (5 mg/ml) solution in phosphate-buffered saline equal to 10% of the culture volume was added to each well and the cells were further incubated for 4 h to form purple MTT formazan crystals. The MTT-containing medium was removed and 200 μL of MTT solvent (0.1 N HCl in anhydrous isopropanol) was added to dissolve MTT crystals. After the cells were gently agitated for 5 min, the absorbance at 540 nm was measured with a BioRad iMark Microplate Reader.

### Cell proliferation assay

UASMC were transfected with siRNA to ZIPK or ROCK1 or with negative control siRNA as described above for 30 h. The medium was replaced with fresh complete medium (containing 5% FBS) and the cells were cultured for a further 18 h. 5-bromo-2-deoxyuridine (BrDU; 10 μM) was then added to the culture medium and the cells were cultured for an additional 1 h. The cells on coverslips were fixed with 10% formalin in neutral buffer (Sigma) and BrDU was detected with Alexa Fluor 488-conjugated anti-BrDU antibody (Invitrogen).

### Enzyme-linked immunosorbent assay (ELISA)

IL-6 levels were quantified in culture supernatants of CASMC and UASMC 48 h after siRNA transfection using an ELISA kit (Human IL-6 DuoSet, DY206, R&D Systems) according to the manufacturer’s instructions. Levels of other cytokines (IL-1α, IP-10 (CXCL10), I-TAC (CXCL11), eotaxin (CCL11), MCP1 (CCL2) and GROα (CXCL1)) were quantified in culture supernatants of CASMC using a Human Custom Multi-Analyte ELISArray kit purchased from Qiagen (CELISA-CMEH0590A). The absorbance was measured at 450 nm with a BioRad iMark Microplate Reader.

### Quantitative real-time PCR (qRT-PCR)

Single-stranded cDNA was synthesized with random hexamer primers using the Superscript II reverse transcriptase according to the manufacturer’s instructions (Invitrogen). Levels of mRNA expression were quantified by RT-PCR using a BioRad iCycler MyiQ detection system (BioRad) with pre-synthesized primers (Applied Biosystems) for ZIPK (Hs00154676_m1), IL-1β (Hs00174097_m1) and IL-6 (Hs00174131_m1). Reactions, which were performed in a MicroAmp Optical 96-well reaction plate, contained: 1x Master Mix, 200 μM each primer, and 100 μM probe in a volume of 25 μL. PCR conditions were 50°C for 2 min, 95°C for 10 min, 40 cycles of 95°C for 15 min/60°C for 1 min. All measurements were performed in duplicate. Expression of gene transcripts was normalized to the housekeeping gene glyceraldehyde-3-phosphate dehydrogenase (GAPDH) [45]. Relative expression levels of the genes of interest were determined using the 2^-ΔΔC^_T_ method [46].

### Western blotting

Cells were lysed directly in 1x sample buffer (65 mM Tris-HCl, pH 6.8, 10% glycerol, 3% SDS, 1% β-mercaptoethanol, 0.04% bromphenol blue) after treatment. Samples were heated at 90°C for 5 min and resolved by SDS-PAGE (11% acrylamide). Proteins were transferred to nitrocellulose membranes in 25 mM Tris, 192 mM glycine, 20% methanol at 30 V overnight at 4°C. Membranes were blocked with 5% non-fat dry milk in 25 mM Tris-HCl, pH 7.4, 150 mM NaCl, 0.05% Tween-20 (TBST) for 1 h at room temperature. Membranes were incubated for 2 h at room temperature with rabbit anti-IL-1β (1:2000; Santa Cruz Biotechnology), rabbit anti-ZIPK (1:1000; Epitomics), rabbit anti-ROCK1 (1:1000; Epitomics), rabbit anti-2P-LC_20_, which recognizes LC_20_ only when phosphorylated at Thr18 and Ser19 (1:500; Cell Signaling), rabbit anti-GAPDH (1:2500; Santa Cruz Biotechnology), goat anti-SM-22 (1:2500; Novus Biologicals) or rabbit anti-α-actin (1:1000; Cytoskeleton, Inc.) in 1% milk in TBST. Membranes were then washed and probed with horseradish peroxidase-conjugated anti-rabbit or anti-goat secondary antibodies (1:100,000 dilution) in 1% milk in TBST. Following three washes with TBST, blots were developed with Supersignal West Femto enhanced chemiluminescence substrate (Thermo Scientific). The emitted light was detected and quantified with a chemiluminescence imaging analyzer (LAS3000mini; Fujifilm) and images were analyzed with MultiGauge version 3.0 software (Fujifilm). Protein levels were expressed relative to the standard, GAPDH, and sometimes to α-actin or SM-22.

### Data analysis

Array data files were generated with GeneChip Command Console Software (AGCC). Quality controls were performed using Expression Console (Affymetrix). Affymetrix raw gene array data were processed using Partek Genomics Suite Software (Partek, Inc.). Two-group comparison was based on *p* < 0.05 (no correction) and fold change ≥ 2.0. Lists of genes showing significant differences in expression levels between groups were subjected to Ingenuity Pathway Analysis (Ingenuity^®^ Systems, www.ingenuity.com) for canonical pathways and network analyses.

To identify genes significantly altered by ROCK1 or ZIPK knockdown, Student’s *t*-test was performed for each assay group. To adjust for multiple hypothesis testing, the resulting *p* values were used to obtain the false discovery rates (FDR) using the *q* value method. Gene expression levels were considered significantly different with FDRs of < 5% (i.e. *q* values ≤ 0.05).

In order to categorize biological functions related to gene expression altered by kinase knockdown in our microarray analysis, Ingenuity Pathway Analysis (IPA, Ingenuity Systems, www.ingenuity.com) was used. The differentially expressed genes (DEGs) identified through the statistical analysis described above (fold-change 1.5 and *q* ≤ 0.05) in both knockdown assays were uploaded into IPA analysis.

Otherwise, statistically significant differences were determined by Student’s unpaired *t*-test at a nominal significance of p < 0.05 and (where applicable) a fold-change in expression > 1.5. ANOVA followed by Bonferroni’s *post hoc* test was used for multiple comparisons.

## Results

### ROCK1 and ZIPK knockdown

**Fig 1:**
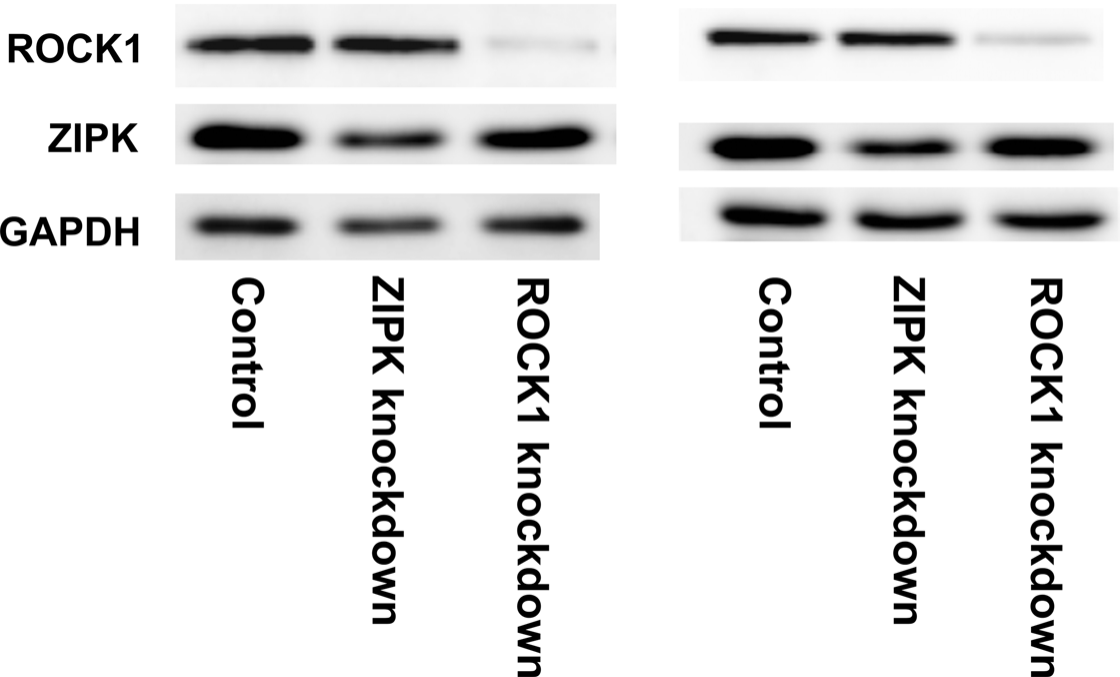
Knockdown of ROCK1 and ZIPK at the protein level in CASMC. CASMC were transfected with siRNAs to ROCK1 or ZIPK or with negative control siRNAs. Cells were lysed 48 h later for western blotting with anti-ROCK1 and anti-ZIPK. Loading levels were normalized using anti-GAPDH. Representative results are shown for two of a total of 10 independent experiments. Quantitative data are presented in Table 2.

The efficiency of siRNA-mediated knockdown of ROCK1 and ZIPK in CASMC was evaluated at both mRNA and protein levels by microarray analysis and western blotting, respectively. ROCK1-targeted siRNA decreased the ROCK1 mRNA level ~12-fold without significantly affecting ZIPK mRNA expression, while ZIPK-targeted siRNA reduced the ZIPK mRNA level >2.5-fold without significantly affecting the ROCK1 mRNA level (Table 1). The reduction in ZIPK mRNA in cells transfected with ZIPK siRNA was confirmed by qRT-PCR (Fig. A in S1 File). At the protein level, ROCK1-targeted siRNA reduced ROCK1 protein expression by ~80% and ZIPK-targeted siRNA reduced ZIPK protein expression by ~50% (Fig. 1 and Table 2). ROCK1 siRNA had no effect on ZIPK protein expression, but ZIPK siRNA treatment was accompanied by a small increase (~30%) in ROCK1 protein expression (Table 2). We also examined the effect of ROCK1 knockdown on ROCK2 expression and found that ROCK1 knockdown (by 80% at the protein level) decreased ROCK2 protein expression to 65.2 ± 9.1% of control (*n* = 6).

**Table 1:** ROCK1 and ZIPK knockdown in CASMC at the mRNA level.

	ROCK1 knockdown		ZIPK knockdown	
Kinase	Fold change	*p* value	Fold change	*p* value
ROCK1	-11.8	1.15 × 10^-5^	-1.01	0.95
ZIPK	1.11	0.53	-2.73	3.5 × 10^-3^

CASMC were transfected with siRNAs targeting ROCK1 or ZIPK or with negative control siRNA as described in the Materials and Methods section. The efficiency of knockdown at the mRNA level was determined by microarray analysis. Values are expressed as fold-change relative to control (*n* = 3).

**Table 2:** ROCK1 and ZIPK knockdown in CASMC at the protein level.

Kinase	ROCK1 knockdown	ZIPK knockdown
ROCK1	0.19 ± 0.07*	1.32 ± 0.15*
ZIPK	1.00 ± 0.30	0.49 ± 0.07*

CASMC were transfected with siRNAs targeting ROCK1 or ZIPK or with negative control siRNA as described in the Materials and Methods section. The efficiency of knockdown at the protein level was determined by western blotting as shown in Fig. 1. ROCK1 and ZIPK signals were normalized to GAPDH. Values are expressed relative to levels in control cells (means ± SD, *n* = 10).

*significantly different from control (*p* < 0.001).

Very similar effects were seen in the case of UASMC, in which the efficiency of siRNA-mediated knockdown was evaluated at the protein level by western blotting. ROCK1-targeted siRNA again reduced ROCK1 protein expression by ~80% and ZIPK-targeted siRNA reduced ZIPK protein expression by ~50% (Table A in S1 File). ROCK1 siRNA again had no effect on ZIPK protein expression, but ZIPK siRNA treatment was accompanied by a small increase (~30%) in ROCK1 protein expression (Table A in S1 File).

### Changes in gene expression profiles induced by ROCK1 and ZIPK knockdown

**Fig 2:**
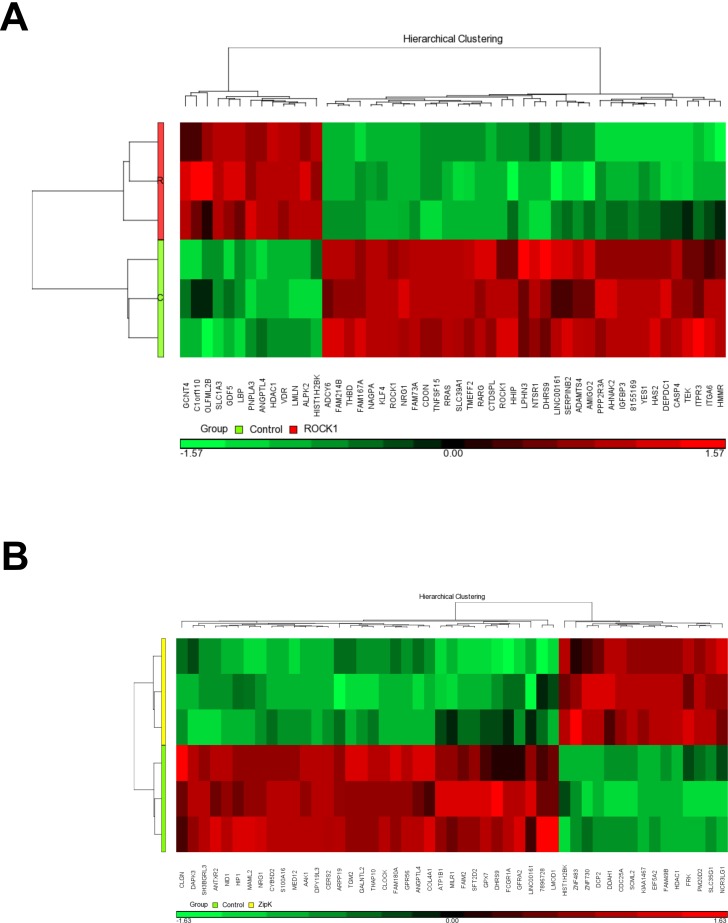
Gene expression profiles. Heat maps to indicate the most differentially expressed genes in (A) ROCK1 knockdown (> 2.4-fold change) and (B) ZIPK knockdown (> 2-fold change) CASMC. Colored bands represent the change of the indicated gene expression: down-regulation green and up-regulation red. The key to decipher the color is shown below the clustering image. Results from the triplicate analyses are included.

Three independent microarray assays were performed on CASMC 48 h after transfection with control, ROCK1 or ZIPK siRNAs using the GeneChip Human Gene 1.0ST array (28,868 genes represented). After normalization through the preprocessing module, a 3D Principal Components Analysis Plot showed clear separation of the data for ROCK1 / ZIPK knockdown from the negative control. Differentially expressed genes (DEGs) were identified on the basis of standard and advanced quality control metrics (FC ≥ 1.5 and *q* values ≤ 0.05); complete lists of these genes are provided in Tables B and C in S1 File for ROCK1 knockdown and ZIPK knockdown, respectively. ROCK1 knockdown affected the expression of 553 genes (355 down-regulated and 198 up-regulated), while ZIPK knockdown affected the expression of 390 genes (219 down-regulated and 171 up-regulated). The gene expression profiles of the most affected genes are shown as heat maps in Fig. 2A (ROCK1 knockdown) and B (ZIPK knockdown).

The DEGs were divided into different functional groups (iReport from http://www.ingenuity.com) (Table 3). A high incidence of up- and down-regulation of transcription regulator genes was observed in both ROCK1 and ZIPK knockdowns. Other markedly affected groups included transporters, kinases, peptidases, transmembrane and G protein-coupled receptors, with multiple genes encoding growth factors, phosphatases and ion channels also being significantly affected. In each of these subgroups, more genes were down-regulated than up-regulated, in contrast to the sum total of affected genes. In the case of the cytokine subgroup, ROCK1 knockdown resulted in up-regulation of 5 genes and down-regulation of 4 genes, in contrast to ZIPK knockdown, which affected the expression of only two genes (both down-regulated).

**Table 3:** The number of DEGs (FC ≥1.5, q-values ≤0.05) in CASMC following knockdown of ROCK1 or ZIPK, and their classification into functional groups.

	ROCK1 knockdown			ZIPK knockdown		
	Total	Up-regulated	Down-regulated	Total	Up-regulated	Down-regulated
**All DEGs**	553	198	355	390	171	219
**Transcription regulators**	31	17	14	35	18	17
**Transporters**	31	7	24	23	7	16
**Kinases**	34	13	21	21	9	12
**Peptidases**	21	6	15	9	2	7
**Transmembrane receptors**	15	4	11	13	1	12
**G protein-coupled receptors**	19	3	16	7	0	7
**Growth factors**	12	3	9	5	0	5
**Phosphatases**	11	3	8	6	2	4
**Ion channels**	11	3	8	6	0	6
**Cytokines**	9	5	4	2	0	2
**Translation regulators**	6	2	4	1	1	0
**Ligand-dependent nuclear receptors**	6	0	6	1	0	1
**microRNAs**	3	3	0	0	0	0
**Enzymes**	93	27	66	62	30	32
**Other**	251	102	149	199	101	98

To gain insight into potential overlapping roles of ROCK1 and ZIPK in vascular SMC, we selected the DEGs that were common to ROCK1 and ZIPK knockdown. There were 76 such DEGs, of which 41 were down-regulated (italics in Table 4) and 26 up-regulated (bold in Table 4) by both treatments, while the other 9 genes were differentially up/down-regulated (bold italics in Table 4). It is clear that many more genes were uniquely affected by ROCK1 or ZIPK knockdown.

**Table 4:** Differentially expressed genes common to ROCK1 and ZIPK knockdown.

Gene Symbol: Gene Name	Fold change		Molecular Function	Location
	ROCK1 knockdown	ZIPK knockdown		
*ADAMTS4*: *ADAM metallopeptidase with thrombospondin type 1 motif*, *4*	*-3.59*	*-2.154*	*peptidase*	*Extracellular Space*
*AGTR1*: *angiotensin II receptor*, *type 1*	*-1.664*	*-1.556*	*G-protein coupled receptor*	*Plasma Membrane*
*AHNAK2*: *AHNAK nucleoprotein 2*	*-2.538*	*-1.677*	*other*	*Unknown*
*DHRS9*: *dehydrogenase/reductase (SDR family) member 9*	*-2.818*	*-2.163*	*enzyme*	*Cytoplasm*
*APOLD1*: *apolipoprotein L domain containing 1*	*-1.67*	*-1.685*	*other*	*Unknown*
*AREG/AREGB*: *amphiregulin*	*-2.139*	*-1.72*	*growth factor*	*Extracellular Space*
*ATP1B1*: *ATPase*, *Na+/K+ transporting*, *beta 1 polypeptide*	*-1.607*	*-2.213*	*transporter*	*Plasma Membrane*
*CERS2*: *ceramide synthase 2*	*-1.761*	*-2.146*	*transcription regulator*	*Nucleus*
*C10orf54*: *chromosome 10 open reading frame 54*	*-1.684*	*-1.688*	*other*	*Unknown*
*FABP3*: *fatty acid binding protein 3*, *muscle and heart (mammary-derived growth inhibitor)*	*-2.127*	*-1.799*	*transporter*	*Cytoplasm*
*FAM180A*: *family with sequence similarity 180*, *member A*	*-1.594*	*-2.722*	*other*	*Unknown*
*FAM214B*: *family with sequence similarity 214*, *member B*	*-2.643*	*-1.51*	*other*	*Nucleus*
*FCGR1A*: *Fc fragment of IgG*, *high affinity Ia*, *receptor (CD64)*	*-1.542*	*-1.863*	*transmembrane receptor*	*Plasma Membrane*
*FCGR1B*: *Fc fragment of IgG*, *high affinity Ib*, *receptor (CD64)*	*-1.562*	*-1.96*	*transmembrane receptor*	*Plasma Membrane*
*FSTL3*: *follistatin-like 3 (secreted glycoprotein)*	*-1.579*	*-1.829*	*other*	*Extracellular Space*
*GJA1*: *gap junction protein*, *alpha 1*, *43kDa*	*-1.707*	*-1.663*	*transporter*	*Plasma Membrane*
*GPR1*: *G protein-coupled receptor 1*	*-2.097*	*-1.619*	*G-protein coupled receptor*	*Plasma Membrane*
*GPR4*: *G protein-coupled receptor 4*	*-1.508*	*-1.617*	*G-protein coupled receptor*	*Plasma Membrane*
*GPRC5A*: *G protein-coupled receptor*, *family C*, *group 5*, *member A*	*-1.634*	*-1.631*	*G-protein coupled receptor*	*Plasma Membrane*
*HHIP*: *hedgehog interacting protein*	*-2.427*	*-1.6*	*other*	*Plasma Membrane*
*ITGA10*: *integrin*, *alpha 10*	*-1.5*	*-1.826*	*other*	*Plasma Membrane*
*JAG1*: *jagged 1*	*-1.61*	*-1.664*	*growth factor*	*Extracellular Space*
*KCND1*: *potassium voltage-gated channel*, *Shal-related subfamily*, *member 1*	*-1.777*	*-1.799*	*ion channel*	*Plasma Membrane*
*KLHL41*: *kelch-like family member 41*	*-1.894*	*-2.146*	*other*	*Cytoplasm*
*LINC00161*: *long intergenic non-protein coding RNA 161*	*-2.414*	*-2.318*	*other*	*Unknown*
*LYVE1*: *lymphatic vessel endothelial hyaluronan receptor 1*	*-1.99*	*-1.887*	*transmembrane receptor*	*Plasma Membrane*
*MALL*: *mal*, *T-cell differentiation protein-like*	*-1.98*	*-1.583*	*other*	*Plasma Membrane*
*MASP1*: *mannan-binding lectin serine peptidase 1 (C4/C2 activating component of Ra-reactive factor)*	*-1.555*	*-1.722*	*peptidase*	*Extracellular Space*
*NDRG1*: *N-myc downstream regulated 1*	*-1.851*	*-1.565*	*kinase*	*Nucleus*
*NR1D2*: *nuclear receptor subfamily 1*, *group D*, *member 2*	*-1.737*	*-1.518*	*ligand-dependent nuclear receptor*	*Nucleus*
*PAQR5*: *progestin and adipoQ receptor family member V*	*-2.27*	*-1.87*	*other*	*Unknown*
*PTGS2*: *prostaglandin-endoperoxide synthase 2 (prostaglandin G/H synthase and cyclooxygenase)*	*-1.733*	*-2.008*	*enzyme*	*Cytoplasm*
*RNF13*: *ring finger protein 13*	*-1.823*	*-1.688*	*enzyme*	*Nucleus*
*S100A16*: *S100 calcium binding protein A16*	*-2.173*	*-2.212*	*other*	*Nucleus*
*SCGB3A2*: *secretoglobin*, *family 3A*, *member 2*	*-1.938*	*-1.897*	*other*	*Extracellular Space*
*SERTAD4*: *SERTA domain containing 4*	*-1.522*	*-1.657*	*other*	*Unknown*
*SLC7A8*: *solute carrier family 7 (amino acid transporter light chain*, *L system)*, *member 8*	*-1.696*	*-1.972*	*transporter*	*Plasma Membrane*
*ST3GAL1*: *ST3 beta-galactoside alpha-2*,*3-sialyltransferase 1*	*-2.12*	*-1.722*	*enzyme*	*Cytoplasm*
*THBD*: *thrombomodulin*	*-4.256*	*-1.611*	*transmembrane receptor*	*Plasma Membrane*
*TNFSF15*: *tumor necrosis factor (ligand) superfamily*, *member 15*	*-2.393*	*-1.78*	*cytokine*	*Extracellular Space*
*TNXB*: *tenascin XB*	*-1.885*	*-1.666*	*other*	*Extracellular Space*
**CARS2: cysteinyl-tRNA synthetase 2, mitochondrial (putative)**	**1.983**	**1.652**	**enzyme**	**Cytoplasm**
**CDC25A: cell division cycle 25A**	**1.65**	**2.212**	**phosphatase**	**Nucleus**
**CELF2: CUGBP, Elav-like family member 2**	**1.571**	**1.838**	**other**	**Nucleus**
**CEP57: centrosomal protein 57kDa**	**1.545**	**1.503**	**other**	**Cytoplasm**
**DDAH1: dimethylarginine dimethylaminohydrolase 1**	**2.112**	**2.339**	**enzyme**	**Cytoplasm**
**EIF5A2: eukaryotic translation initiation factor 5A2**	**2.193**	**2.044**	**translation regulator**	**Cytoplasm**
**EML4: echinoderm microtubule associated protein like 4**	**1.744**	**1.545**	**other**	**Cytoplasm**
**ENTPD7: ectonucleoside triphosphate diphosphohydrolase 7**	**1.822**	**1.55**	**enzyme**	**Cytoplasm**
**ESCO2: establishment of sister chromatid cohesion N-acetyltransferase 2**	**1.629**	**1.663**	**other**	**Nucleus**
**FAM49B: family with sequence similarity 49, member B**	**2.203**	**2.711**	**other**	**Extracellular Space**
**GNL3L: guanine nucleotide binding protein-like 3 (nucleolar)-like**	**1.65**	**1.559**	**other**	**Nucleus**
**HDAC1: histone deacetylase 1**	**2.514**	**2.613**	**transcription regulator**	**Nucleus**
**HIST1H2BJ/HIST1H2BK: histone cluster 1, H2bk**	**1.963**	**1.694**	**other**	**Nucleus**
**KIAA1467: KIAA1467**	**2.133**	**2.221**	**other**	**Unknown**
**MICB: MHC class I polypeptide-related sequence B**	**1.674**	**1.866**	**transmembrane receptor**	**Plasma Membrane**
**NUP54: nucleoporin 54kDa**	**1.662**	**1.511**	**transporter**	**Nucleus**
**RNF144B: ring finger protein 144B**	**2.02**	**1.596**	**enzyme**	**Unknown**
**SERPINB9: serpin peptidase inhibitor, clade B (ovalbumin), member 9**	**1.893**	**1.942**	**other**	**Cytoplasm**
**SNORD78: small nucleolar RNA, C/D box 78**	**1.507**	**1.505**	**other**	**Unknown**
**SPIN4: spindlin family, member 4**	**1.855**	**1.886**	**other**	**Unknown**
**TMEM144: transmembrane protein 144**	**1.822**	**1.596**	**other**	**Unknown**
**UBE2D1: ubiquitin-conjugating enzyme E2D 1**	**2.117**	**1.83**	**enzyme**	**Cytoplasm**
**VWA9: von Willebrand factor A domain containing 9**	**1.832**	**1.564**	**other**	**Cytoplasm**
**ZC3HAV1L: zinc finger CCCH-type, antiviral 1-like**	**2.304**	**1.907**	**other**	**Unknown**
**ZNF100: zinc finger protein 100**	**1.643**	**1.907**	**other**	**Nucleus**
**AGK: acylglycerol kinase**	**1.56**	**1.722**	**kinase**	**Cytoplasm**
***FBXO27*: *F-box protein 27***	***-1.643***	***1.714***	***other***	***Unknown***
***RAD51AP1*: *RAD51 associated protein 1***	***-1.559***	***1.68***	***other***	***Nucleus***
***ADA*: *adenosine deaminase***	***1.758***	***-1.57***	***enzyme***	***Cytoplasm***
***FAM87B*: *family with sequence similarity 87*, *member B***	***1.692***	***-1.533***	***other***	***Unknown***
***GALNT15*: *UDP-N-acetyl-alpha-D-galactosamine*:*polypeptide N-acetylgalactosaminyltransferase 15***	***1.891***	***-2.351***	***enzyme***	***Cytoplasm***
***ANGPTL4*: *angiopoietin-like 4***	***2.536***	***-2.05***	***other***	***Extracellular Space***
***BMP2K*: *BMP2 inducible kinase***	***1.542***	***-1.868***	***kinase***	***Nucleus***
***C1orf110*: *chromosome 1 open reading frame 110***	***2.595***	***-1.592***	***other***	***Unknown***
***CPA4*: *carboxypeptidase A4***	***1.856***	***-1.653***	***peptidase***	***Extracellular Space***

### Analysis of biological pathways and processes regulated by ROCK1 and ZIPK

**Fig 3:**
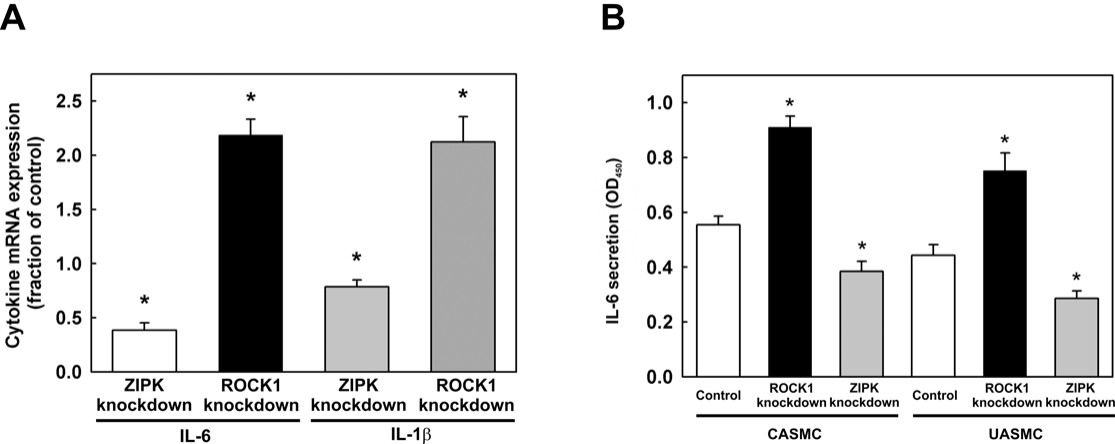
Effects of ROCK1 and ZIPK knockdown on IL-6 and IL-1β mRNA expression and IL-6 protein secretion. (A) CASMCs were transfected with siRNA to ZIPK or ROCK1 or with negative control siRNA. Cells were lysed 48 h later for qRT-PCR to quantify IL-6 and IL-1β mRNA levels. Values represent means ± SEM (*n* = 7 for IL-6 and *n* = 6 for IL-1β). *significantly different from control (p < 0.001 except for IL-1β in ZIPK knockdown where *p* = 0.007). (B) IL-6 levels in the medium of control cells and cells transfected with ROCK1 or ZIPK siRNA were quantified by ELISA. Values represent means ± SEM (*n* = 20 for CASMC, *n* = 29 for UASMC). *significantly different from control (*p* < 0.002).

Ingenuity Pathway Analysis (IPA; Ingenuity Systems, www.ingenuity.com) was used to facilitate the identification of biological themes in the microarray data. IPA shifts the emphasis from the evaluation of single genes to an evaluation of molecular pathways, networks and biological functions. 72 canonical pathways with *p* values < 0.05 were affected by ROCK1 knockdown (Table 5) and 27 by ZIPK knockdown (Table 6). Most interestingly, ROCK1 knockdown affected several cytokine signaling pathways. For example, IL-6 gene expression was up-regulated by ROCK1 knockdown (2.24-fold; *p* = 0.015) according to the microarray analysis (Table 5), and this was confirmed by qRT-PCR (Fig. 3A) and quantification of IL-6 protein levels in the medium of CASMC and UASMC transfected with ROCK1 siRNA (Fig. 3B). ZIPK knockdown, on the other hand, reduced IL-6 mRNA expression (Fig. 3A) and IL-6 protein secretion (Fig. 3B). qRT-PCR also demonstrated no significant changes in expression level of the housekeeping gene GAPDH as a result of ROCK1 or ZIPK knockdown (data not shown). These results suggest that ROCK1 may inhibit IL-6 secretion. It has been reported that IL-6 promotes tumor cell migration and invasion, and that ROCK1 may be involved in this process [47]. Furthermore, since ZIPK knockdown results in increased ROCK1 protein expression (Fig. 1, Table 2, and Table A in S1 File), the observed decrease in IL-6 secretion following transfection with ZIPK siRNA may be due to increased ROCK1 expression. This possibility was supported by the effects of combined knockdown of ROCK1 and ZIPK. The combination of ZIPK-siRNA and ROCK1-siRNA reduced ZIPK protein levels to 64.9% of control and ROCK1 levels to 64.1% of control. The combined knockdown of ZIPK and ROCK1 had an intermediate effect on IL-6 secretion compared with knockdown of ZIPK and ROCK1 individually, i.e. IL-6 increased by 47.8 ± 13.4% (*n* = 9) in the double-knockdown compared with a decrease in IL-6 secretion in ZIPK-knockdown cells to 59.8 ± 5.5% of control siRNA-treated cells (*n* = 9) and an increase in IL-6 secretion by 143.6 ± 11.4% (*n* = 9) in ROCK1-knockdown cells. These results support the conclusion that some genes are regulated by both kinases and that up-regulation of ROCK1 partially compensates for reduced ZIPK expression.

**Table 5:** Top 72 pathways generated by IPA from DEGs regulated by ROCK1 knockdown (of 368 with *p* < 0.05).

Pathway	DEGs	*p* value	Genes
**Cell Cycle: G1/S Checkpoint Regulation**	**8**	**0.000265**	**HDAC1, CCNE2, CDC25A, MYC, CDKN2B, CCND3, NRG1, BTRC**
*Role of JAK family kinases in IL-6-type Cytokine Signaling*	*5*	*0.000496*	*IL6*, *SOCS3*, *STAT1*, *MAPK10*, *MAPK9*
*Endothelin-1 Signaling*	*13*	*0.000834*	*PNPLA3*, *RARRES3*, *MYC*, *PLCD1*, *MAPK10*, *MAPK9*, *EDNRB*, *GNA14*, *PTGS2*, *ITPR3*, *ADCY6*, *CASP4*, *RRAS*
*GNRH Signaling*	*11*	*0.00086*	*MAPK10*, *PRKAR1A*, *MAP3K3*, *MAPK9*, *CAMK2D*, *GNA14*, *PRKACA*, *MAP3K2*, *ITPR3*, *ADCY6*, *RRAS*
*Role of Tissue Factor in Cancer*	*10*	*0.000903*	*CYR61*, *CTGF*, *F2RL1*, *CFL1*, *VEGFC*, *FGF5*, *GNA14*, *ITGA6*, *YES1*, *RRAS*
*Prostanoid Biosynthesis*	*3*	*0.001491*	*PTGS2*, *PTGES*, *PTGIS*
*Interferon Signaling*	*5*	*0.002125*	*IFI35*, *STAT1*, *IFITM1*, *IRF1*, *IFNAR1*
*CDK5 Signaling*	*8*	*0.00273*	*CAPN1*, *PRKAR1A*, *ITGA2*, *PRKACA*, *PPP2R3A*, *ITGA6*, *ADCY6*, *RRAS*
*Renin-Angiotensin Signaling*	*9*	*0.002938*	*RRAS*, *ITPR3*, *PRKACA*, *ADCY6*, *MAPK10*, *MAPK9*, *STAT1*, *AGTR1*, *PRKAR1A*
*IL-22 Signaling*	*4*	*0.003743*	*SOCS3*, *STAT1*, *MAPK10*, *MAPK9*
*Role of NFAT in Cardiac Hypertrophy*	*12*	*0.004255*	*HDAC1*, *IL6*, *PLCD1*, *MAPK10*, *PPP3R1*, *PRKAR1A*, *MAPK9*, *CAMK2D*, *PRKACA*, *ITPR3*, *ADCY6*, *RRAS*
*Role of Macrophages*, *Fibroblasts and Endothelial Cells in Rheumatoid Arthritis*	*17*	*0.004343*	*IL6*, *SOCS3*, *TNFRSF11B*, *CEBPD*, *MYC*, *PLCD1*, *TNFSF13B*, *F2RL1*, *FCGR1A*, *PPP3R1*, *WNT16*, *MAPK9*, *CAMK2D*, *VEGFC*, *ADAMTS4*, *RRAS*, *ROCK1*
*Hepatic Fibrosis / Hepatic Stellate Cell Activation*	*10*	*0.004773*	*CTGF*, *EDNRB*, *IGFBP3*, *VEGFC*, *IL6*, *LBP*, *STAT1*, *AGTR1*, *IFNAR1*, *TNFRSF11B*
*Dopamine-DARPP32 Feedback in cAMP Signaling*	*11*	*0.005113*	*PLCD1*, *KCNJ2*, *PPP2R3A*, *PPP3R1*, *ITPR3*, *CREM*, *PRKACA*, *ADCY6*, *PAWR*, *KCNJ6*, *PRKAR1A*
**Cyclins and Cell Cycle Regulation**	**7**	**0.006103**	**CCNE2, CCND3, PPP2R3A, HDAC1, BTRC, CDKN2B, CDC25A**
*Eicosanoid Signaling*	*6*	*0.006701*	*PNPLA3*, *RARRES3*, *CYSLTR1*, *PTGS2*, *PTGES*, *PTGIS*
**The Visual Cycle**	**3**	**0.007153**	**DHRS3, RDH11, DHRS9**
*Axonal Guidance Signaling*	*21*	*0.007242*	*NTF3*, *SHANK2*, *PLCD1*, *ADAMTS1*, *PPP3R1*, *PRKAR1A*, *ITGA2*, *ADAMTS5*, *CFL1*, *WNT16*, *VEGFC*, *GNA14*, *ADAMTS6*, *PRKACA*, *BMP6*, *SEMA3A*, *PLXNA2*, *HHIP*, *ADAMTS4*, *RRAS*, *ROCK1*
*RAR Activation*	*11*	*0.008237*	*CRABP2*, *MAPK10*, *SNW1*, *PRKAR1A*, *MAPK9*, *PRKACA*, *RDH11*, *IGFBP3*, *ADCY6*, *DHRS9*, *RARG*
*Cardiac Hypertrophy Signaling*	*13*	*0.008594*	*IL6*, *PLCD1*, *MAPK10*, *PPP3R1*, *PRKAR1A*, *MAP3K3*, *MAPK9*, *GNA14*, *PRKACA*, *MAP3K2*, *ADCY6*, *RRAS*, *ROCK1*
*Role of MAPK Signaling in the Pathogenesis of Influenza*	*6*	*0.009715*	*PNPLA3*, *RARRES3*, *MAPK10*, *MAPK9*, *PTGS2*, *RRAS*
*Retinoic acid Mediated Apoptosis Signaling*	*6*	*0.009715*	*CRABP2*, *TNFSF10*, *PARP9*, *RARG*, *IRF1*, *IFNAR1*
*Agrin Interactions at Neuromuscular Junction*	*6*	*0.010417*	*MAPK10*, *ITGA2*, *MAPK9*, *NRG1*, *ITGA6*, *RRAS*
*Amyloid Processing*	*5*	*0.012312*	*CAPN1*, *PRKAR1A*, *APH1B*, *CAPN5*, *PRKACA*
**Molecular Mechanisms of Cancer**	**17**	**0.012542**	**CCNE2, CDC25A, MYC, CDKN2B, MAPK10, PRKAR1A, APH1B, MAPK9, CAMK2D, LAMTOR3, GNA14, CCND3, PRKACA, BMP6, BIRC3, ADCY6, RRAS**
*D-myo-inositol-5-phosphate Metabolism*	*9*	*0.012742*	*SOCS3*, *CDC25A*, *PAWR*, *CCR1*, *PLCD1*, *PTPN22*, *PTPRM*, *PPTC7*, *PPP2R3A*
*IL-17 Signaling*	*6*	*0.013588*	*IL6*, *CCL11*, *MAPK10*, *MAPK9*, *PTGS2*, *RRAS*
*RhoA Signaling*	*8*	*0.014708*	*CDC42EP3*, *LPAR6*, *ARHGAP12*, *CFL1*, *RAPGEF6*, *PIKFYVE*, *CIT*, *ROCK1*
*Colorectal Cancer Metastasis Signaling*	*13*	*0.015609*	*IL6*, *STAT1*, *MYC*, *MAPK10*, *PRKAR1A*, *WNT16*, *MAPK9*, *VEGFC*, *PTGS2*, *MMP16*, *PRKACA*, *ADCY6*, *RRAS*
*Leptin Signaling in Obesity*	*6*	*0.016369*	*SOCS3*, *PLCD1*, *PRKAR1A*, *PDE3A*, *PRKACA*, *ADCY6*
*BMP signaling pathway*	*6*	*0.016369*	*MAPK10*, *PRKAR1A*, *MAPK9*, *PRKACA*, *BMP6*, *RRAS*
*Nur77 Signaling in T Lymphocytes*	*5*	*0.016699*	*HDAC1*, *PPP3R1*, *MAP3K3*, *MAP3K2*, *NR4A1*
*IGF-1 Signaling*	*7*	*0.0167*	*SOCS3*, *CYR61*, *CTGF*, *PRKAR1A*, *PRKACA*, *IGFBP3*, *RRAS*
*Synaptic Long Term Potentiation*	*8*	*0.016905*	*PLCD1*, *PPP3R1*, *PRKAR1A*, *CAMK2D*, *GNA14*, *PRKACA*, *ITPR3*, *RRAS*
*HGF Signaling*	*7*	*0.019431*	*IL6*, *MAPK10*, *MAP3K3*, *MAPK9*, *PTGS2*, *MAP3K2*, *RRAS*
*Granulocyte Adhesion and Diapedesis*	*10*	*0.020295*	*CCL7*, *TNFRSF11B*, *SDC4*, *CCL8*, *CCL11*, *ICAM2*, *ITGA2*, *SDC3*, *MMP16*, *ITGA6*
*Cholecystokinin/Gastrin-mediated Signaling*	*7*	*0.020407*	*CREM*, *MAPK10*, *MAPK9*, *PTGS2*, *ITPR3*, *RRAS*, *ROCK1*
*Reelin Signaling in Neurons*	*6*	*0.020656*	*MAPK10*, *ITGA2*, *MAPK9*, *VLDLR*, *ITGA6*, *YES1*
*Pyrimidine Ribonucleotides Interconversion*	*3*	*0.021037*	*ENTPD7*, *NME5*, *ENTPD3*
*Polyamine Regulation in Colon Cancer*	*3*	*0.021037*	*PSME2*, *MYC*, *PPARG*
*Role of PKR in Interferon Induction and Antiviral Response*	*4*	*0.022906*	*STAT1*, *IRF1*, *FCGR1A*, *RNASEL*
*Thyroid Cancer Signaling*	*4*	*0.022906*	*MYC*, *PPARG*, *NTF3*, *RRAS*
*Dendritic Cell Maturation*	*10*	*0.024166*	*IL6*, *TNFRSF11B*, *STAT4*, *STAT1*, *PLCD1*, *MAPK10*, *FCGR1A*, *FCGR1B*, *MAPK9*, *IFNAR1*
**Estrogen-mediated S-phase Entry**	**3**	**0.026568**	**CCNE2, CDC25A, MYC**
*Pyrimidine Ribonucleotides De Novo Biosynthesis*	*3*	*0.026568*	*ENTPD7*, *NME5*, *ENTPD3*
*Adenine and Adenosine Salvage VI*	*1*	*0.027232*	*ADK*
*GM-CSF Signaling*	*5*	*0.028326*	*STAT1*, *PIM1*, *PPP3R1*, *CAMK2D*, *RRAS*
*Activation of IRF by Cytosolic Pattern Recognition Receptors*	*5*	*0.028326*	*IL6*, *STAT1*, *MAPK10*, *MAPK9*, *IFNAR1*
*ERK5 Signaling*	*5*	*0.030065*	*MYC*, *MAP3K3*, *WNK1*, *MAP3K2*, *RRAS*
*RANK Signaling in Osteoclasts*	*6*	*0.031381*	*MAPK10*, *PPP3R1*, *MAP3K3*, *MAPK9*, *MAP3K2*, *BIRC3*
*Superpathway of Inositol Phosphate Compounds*	*10*	*0.031425*	*SOCS3*, *CDC25A*, *PAWR*, *CCR1*, *PLCD1*, *PTPN22*, *PTPRM*, *PIKFYVE*, *PPTC7*, *PPP2R3A*
*Neuregulin Signaling*	*6*	*0.032935*	*MYC*, *ITGA2*, *NRG1*, *AREG/AREGB*, *TMEFF2*, *RRAS*
*Bladder Cancer Signaling*	*6*	*0.034538*	*MYC*, *FGF10*, *RRAS*, *MMP16*, *VEGFC*, *FGF5*
*3-phosphoinositide Degradation*	*8*	*0.035227*	*SOCS3*, *INPP4B*, *PPP2R3A*, *PPTC7*, *PAWR*, *PTPRM*, *PTPN22*, *CDC25A*
*JAK/Stat Signaling*	*5*	*0.035678*	*IL6*, *SOCS3*, *STAT4*, *STAT1*, *RRAS*
*IL-15 Production*	*3*	*0.036173*	*IL6*, *STAT1*, *IRF1*
*D-myo-inositol (1*,*4*,*5)-Trisphosphate Biosynthesis*	*3*	*0.036173*	*CCR1*, *PLCD1*, *PIKFYVE*
*Hepatic Cholestasis*	*8*	*0.036529*	*PRKACA*, *ADCY6*, *MAPK10*, *MAPK9*, *IL6*, *LBP*, *PRKAR1A*, *TNFRSF11B*
*UVA-Induced MAPK Signaling*	*6*	*0.037893*	*PLCD1*, *RRAS*, *MAPK10*, *MAPK9*, *STAT1*, *PARP9*
*Role of Osteoblasts*, *Osteoclasts and Chondrocytes in Rheumatoid Arthritis*	*11*	*0.037921*	*IL6*, *TNFRSF11B*, *MAPK10*, *PPP3R1*, *ITGA2*, *ADAMTS5*, *WNT16*, *MAPK9*, *BMP6*, *BIRC3*, *ADAMTS4*
*NF-κB Signaling*	*9*	*0.038212*	*HDAC1*, *TNFRSF11B*, *TNFSF13B*, *MAP3K3*, *UBE2N*, *PRKACA*, *GHR*, *BTRC*, *RRAS*
*cAMP-mediated signaling*	*11*	*0.038985*	*NPR3*, *CREM*, *PPP3R1*, *PRKAR1A*, *CAMK2D*, *LAMTOR3*, *AGTR1*, *PDE3A*, *PRKACA*, *HTR4*, *ADCY6*
*IL-6 Signaling*	*7*	*0.03934*	*LBP*, *IL6*, *SOCS3*, *TNFRSF11B*, *MAPK10*, *MAPK9*, *RRAS*
*Chemokine Signaling*	*5*	*0.039755*	*CCL7*, *CCL11*, *CFL1*, *CAMK2D*, *RRAS*
*Retinol Biosynthesis*	*4*	*0.041226*	*DHRS3*, *DHRS9*, *RDH11*, *PNPLA3*
*IL-1 Signaling*	*6*	*0.041449*	*MAPK10*, *PRKAR1A*, *MAPK9*, *GNA14*, *PRKACA*, *ADCY6*
*Antioxidant Action of Vitamin C*	*6*	*0.047166*	*PLCD1*, *SLC2A1*, *MAPK10*, *MAPK9*, *RARRES3*, *PNPLA3*
*Sonic Hedgehog Signaling*	*3*	*0.047318*	*PRKACA*, *HHIP*, *PRKAR1A*
*D-myo-inositol (3*,*4*,*5*,*6)-Tetrakisphosphate Biosynthesis*	*7*	*0.047582*	*SOCS3*, *PPP2R3A*, *PPTC7*, *PAWR*, *PTPRM*, *PTPN22*, *CDC25A*
*D-myo-inositol (1*,*4*,*5*,*6)-Tetrakisphosphate Biosynthesis*	*7*	*0.047582*	*SOCS3*, *PPP2R3A*, *PPTC7*, *PAWR*, *PTPRM*, *PTPN22*, *CDC25A*
*Prolactin Signaling*	*5*	*0.048727*	*MYC*, *SOCS3*, *RRAS*, *STAT1*, *IRF1*
*CD27 Signaling in Lymphocytes*	*4*	*0.049733*	*MAPK10*, *MAPK9*, *MAP3K3*, *MAP3K2*

**Table 6:** Top 27 pathways generated by IPA from DEGs regulated by ZIPK knockdown (of 302 with *p* < 0.05).

Pathway	DEGs	*p* value	Genes
**Cell Cycle: G1/S Checkpoint Regulation**	**10**	**2.68E-07**	**HDAC1, CDC25A, CCNE1, CDK6, CCNE2, RBL1, SKP2, E2F5, CCND2, TGFB3**
**Cyclins and Cell Cycle Regulation**	**10**	**3.02E-06**	**CCNE1, CCNE2, CCND2, WEE1, HDAC1, TGFB3, CDK6, E2F5, CDC25A, SKP2**
**Estrogen-mediated S-phase Entry**	**6**	**4.86E-06**	**CCNE1, CCNE2, E2F5, RBL1, CDC25A, SKP2**
*Cell Cycle Control of Chromosomal Replication*	*6*	*1.27E-05*	*MCM3*, *MCM5*, *MCM6*, *CDC6*, *CDK6*, *MCM7*
*Antiproliferative Role of TOB in T Cell Signaling*	*5*	*0.00012*	*CCNE1*, *CCNE2*, *TGFB3*, *TOB1*, *SKP2*
*Pancreatic Adenocarcinoma Signaling*	*9*	*0.000222*	*RAD51*, *CCNE1*, *TGFB3*, *MAPK8*, *E2F5*, *HBEGF*, *PTGS2*, *PLD1*, *PGF*
*Aryl Hydrocarbon Receptor Signaling*	*10*	*0.000381*	*TGM2*, *GSTT2/GSTT2B*, *CCNE1*, *CCNE2*, *CCND2*, *TGFB3*, *CDK6*, *APAF1*, *MAPK8*, *MCM7*
*Small Cell Lung Cancer Signaling*	*6*	*0.002424*	*CCNE1*, *CCNE2*, *CDK6*, *APAF1*, *PTGS2*, *SKP2*
*Ceramide Biosynthesis*	*2*	*0.005254*	*SPTLC2*, *SPTLC3*
*Notch Signaling*	*4*	*0.005922*	*MAML2*, *NOTCH3*, *MAML3*, *JAG1*
*GADD45 Signaling*	*3*	*0.006302*	*CCNE1*, *CCNE2*, *CCND2*
*Factors Promoting Cardiogenesis in Vertebrates*	*6*	*0.006997*	*CCNE1*, *CCNE2*, *CDC6*, *TGFB3*, *TCF7*, *FZD7*
*p53 Signaling*	*6*	*0.009091*	*TP53INP1*, *CCND2*, *HDAC1*, *APAF1*, *MAPK8*, *HIPK2*
*Tumoricidal Function of Hepatic Natural Killer Cells*	*3*	*0.010574*	*SERPINB9*, *APAF1*, *LYVE1*
*Ovarian Cancer Signaling*	*7*	*0.014482*	*RAD51*, *GJA1*, *MSH2*, *PTGS2*, *TCF7*, *FZD7*, *PGF*
*Death Receptor Signaling*	*4*	*0.025274*	*TNFRSF21*, *APAF1*, *MAPK8*, *TNFSF15*
*Role of BRCA1 in DNA Damage Response*	*4*	*0.028203*	*RAD51*, *MSH2*, *E2F5*, *RBL1*
*Cell Cycle Regulation by BTG Family Proteins*	*3*	*0.0293*	*CCNE1*, *CCNE2*, *E2F5*
*Coagulation System*	*3*	*0.0293*	*PLAU*, *THBD*, *SERPIND1*
*CDP-diacylglycerol Biosynthesis I*	*2*	*0.03282*	*GPAM*, *AGPAT9*
**The Visual Cycle**	**2**	**0.03282**	**DHRS9, RDH5**
**Molecular Mechanisms of Cancer**	**12**	**0.032875**	**CCNE1, CCNE2, CCND2, TGFB3, CDK6, APAF1, MAPK8, E2F5, HIPK2, RBL1, FZD7, CDC25A**
*Chronic Myeloid Leukemia Signaling*	*5*	*0.034984*	*HDAC1*, *TGFB3*, *CDK6*, *E2F5*, *RBL1*
*Pyridoxal 5′-phosphate Salvage Pathway*	*4*	*0.036378*	*CDK18*, *MAPK6*, *CDK6*, *MAPK8*
*Mismatch Repair in Eukaryotes*	*2*	*0.03704*	*MSH2*, *EXO1*
*Phosphatidylglycerol Biosynthesis II (Non-plastidic)*	*2*	*0.041456*	*GPAM*, *AGPAT9*
*MIF Regulation of Innate Immunity*	*3*	*0.043901*	*TLR4*, *MAPK8*, *PTGS2*

**Fig 4:**
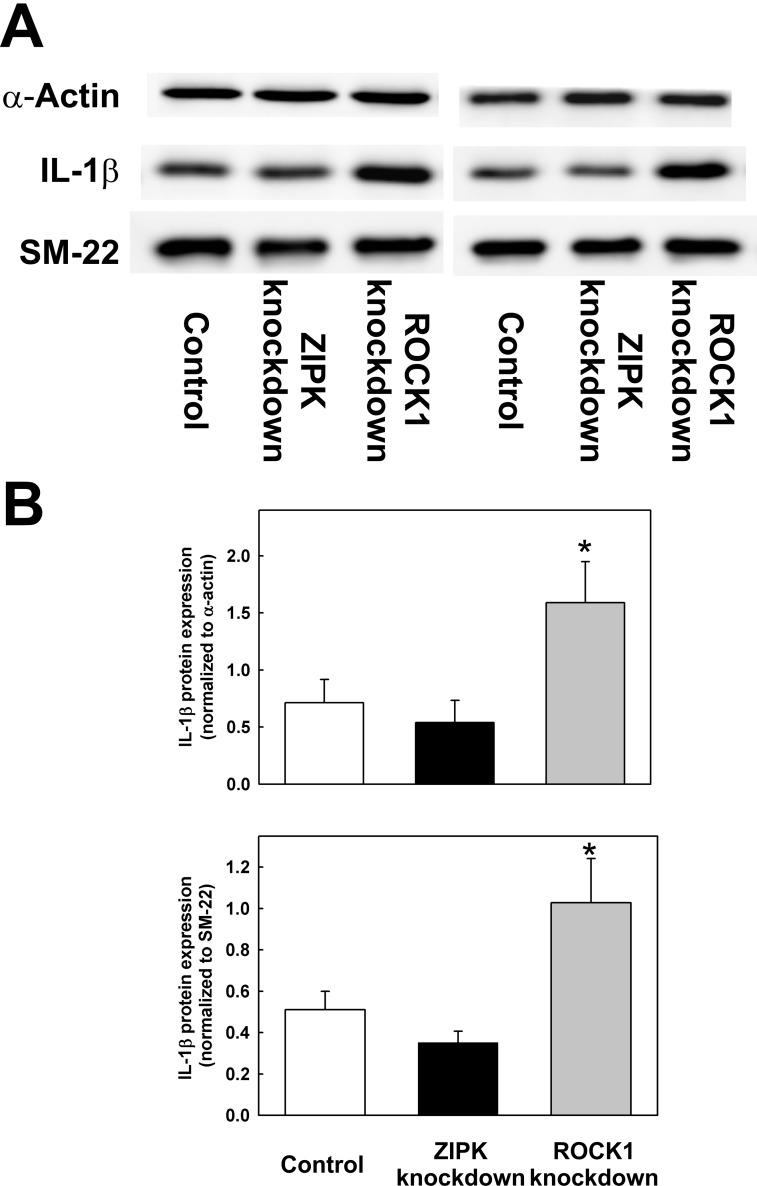
Effects of ROCK1 and ZIPK knockdown on IL-1β protein expression. IL-1β protein expression in control cells and cells transfected with ZIPK or ROCK1 siRNA was examined by western blotting. (A) Two representative western blots showing the levels of IL-1β in control UASMC and in UASMC 48 h after transfection with siRNAs to ZIPK or ROCK1. Two loading controls were used: α-actin and SM-22. (B) Quantification of IL-1β expression levels relative to α-actin (upper panel) and SM-22 (lower panel) in control, ZIPK- and ROCK1-knockdown UASMC. Values represent means ± SEM (*n* = 13 for α-actin and *n* = 9 for SM-22). *significantly different from control (*p* < 0.05). In the case of ZIPK knockdown, statistical significance was not achieved (*p* = 0.54 for α-actin and *p* = 0.15 for SM-22).

IL-1β mRNA (Fig. 3A) and protein levels (Fig. 4) were increased ~2-fold in response to ROCK1 knockdown. ZIPK knockdown slightly reduced IL-1β mRNA expression compared to control (Fig. 3A), but the apparent reduction in IL-1β protein expression was not statistically significant (Fig. 4). We also quantified the levels of several other cytokines in the culture medium of control cells and cells transfected with siRNA to ROCK1 or ZIPK. As shown above for IL-6, MCP1 (CCL2) secretion was increased in cells transfected with siRNA to ROCK1 and decreased in cells transfected with siRNA to ZIPK (Table D in S1 File).

**Fig 5:**
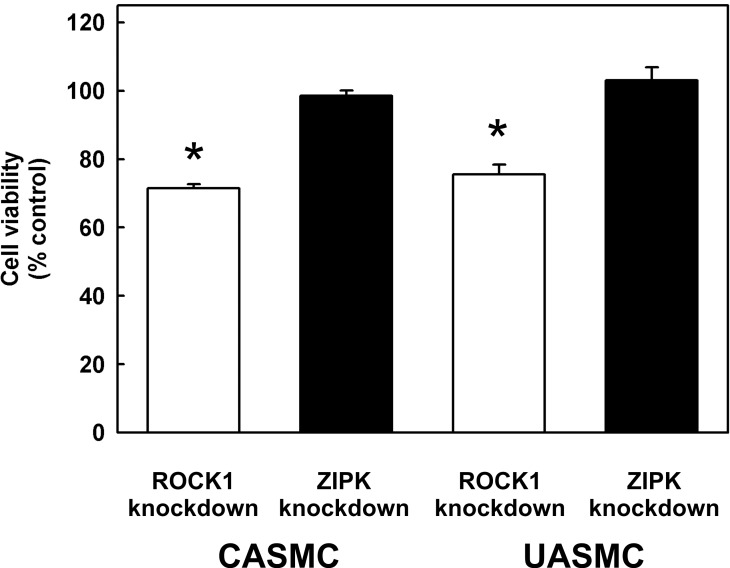
Effects of ROCK1 and ZIPK knockdown on vascular smooth muscle cell viability. The MTT cell viability assay was performed on CASMC and UASMC 48 h after transfection with siRNA to ROCK1 or ZIPK or with negative control siRNA. Values represent the means ± SEM (*n* = 8). *significantly different from control (*p* < 0.001).

Five common pathways were affected by both ROCK1 and ZIPK knockdown (highlighted in bold in Tables 5 and 6) and they are mainly involved in cell cycle regulation. The Cell Cycle: G1/S Checkpoint Regulation pathway contains the largest number of genes whose expression is affected by both ROCK1 and ZIPK knockdown: ROCK1 knockdown resulted in 8 DEGs (4 up-regulated) while ZIPK knockdown resulted in 10 DEGs (8 up-regulated). Three up-regulated genes (HDAC1, CDC25A, and CCNE2) were shared by both ROCK1 and ZIPK knockdowns and all others were differentially up/down-regulated, indicating that ROCK1 and ZIPK regulate the cell cycle by different mechanisms. These differences may account for our observation that ROCK1, but not ZIPK knockdown, significantly reduced the viability of vascular SMC from both coronary and umbilical arteries: the viability of both cell types was reduced (by ~ 25% in the case of CASMC and ~ 29% in the case of UASMC) as a result of ROCK1 knockdown (Fig. 5). ZIPK knockdown, on the other hand, had no significant impact on cell viability when compared to the negative controls.

**Fig 6:**
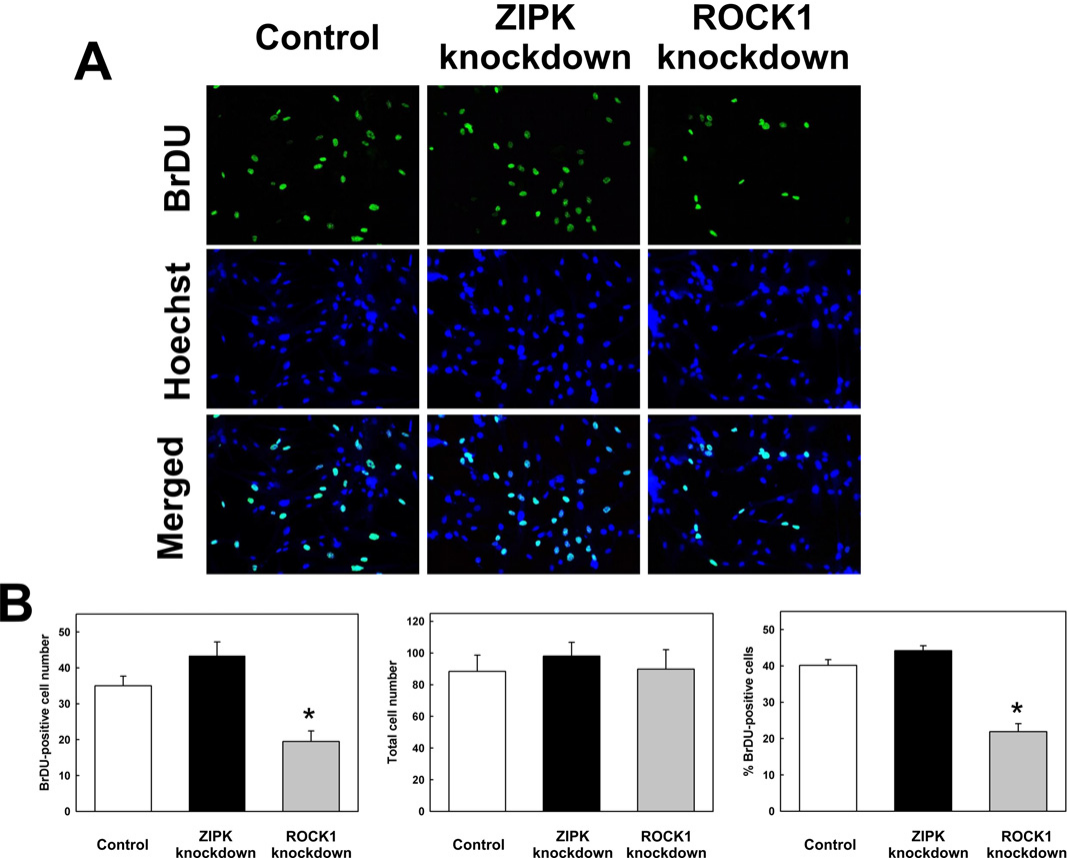
Effects of ROCK1 and ZIPK knockdown on cell proliferation. BrDU incorporation was observed by anti-BrDU staining in UASMC 48 h following transfection with siRNA to ZIPK or ROCK1 or with negative control siRNA. (A) Representative fields of cells are shown stained with anti-BrDU (top panels) or Hoechst nuclear stain (middle panels) with merged images shown in the bottom panels. (B) Quantitative data showing the numbers of BrDU-positive cells (left panel), total numbers of cells (middle panel) and % BrDU-positive cells (right panel). Fields were chosen to contain approximately the same number of cells (as shown in the middle panel) although, as shown by the MTT assay (Fig. 5), ROCK1 knockdown reduced overall cell viability. Values represent means ± SEM (*n* = 4 independent experiments, in each of which 3 fields of cells were counted for control, ZIPK knockdown and ROCK1 knockdown). *significantly different from control (p < 0.01).

The effects of ROCK1 and ZIPK knockdown on UASMC proliferation were investigated by measuring BrDU incorporation 48 h after siRNA transfection. ZIPK knockdown had no significant effect on the proportion of BrDU-positive cells compared with cells transfected with control siRNA (Fig. 6). On the other hand, ROCK1 knockdown reduced the proportion of BrDU-positive cells by ~50% (from 40.2 ± 1.6% to 21.9 ± 2.2% of the total cell population). These results support an important role for ROCK1 in vascular SMC proliferation, as previously proposed [48], and suggest that ZIPK does not affect proliferation. It is important to note that, in the experiments of Fig. 6, fields were selected to contain approximately the same number of cells although, consistent with the MTT assay (Fig. 5), ROCK1 knockdown reduced overall cell viability. Furthermore, we cannot rule out the possibility that the lack of effect of ZIPK knockdown on viability and proliferation may be due to the fact that ~50% of cellular ZIPK is retained.

Apart from classifying individual genes into pathway categories, IPA also predicts corresponding biological processes that are significantly associated with identified DEGs. The main processes associated with the most significantly altered genes in ROCK1-knockdown CASMC were cell migration and movement, apoptosis, tissue development, smooth muscle cell proliferation, cell differentiation and protein phosphorylation (Table 7). The main processes associated with the most significantly altered genes in ZIPK-knockdown CASMC were cell cycle, cell death and viability, necrosis, DNA metabolism and replication (Table 8). Four biological processes (highlighted in bold in Tables 7 and 8) were shared by the two groups: necrosis, apoptosis, proliferation of cells, and cell death of tumor cell lines. Processes unique to ROCK1 knockdown include cell migration and movement, and smooth muscle development, and those unique to ZIPK knockdown include cell cycle and cell viability.

**Table 7:** Top 25 biological processes associated with ROCK1 knockdown (of 333 with *p* < 0.003).

Biological Process	DEGs	*p* value
*migration of cells*	*116*	*2.76E-12*
*cell movement*	*124*	*8.07E-12*
**apoptosis**	**152**	**1.95E-11**
*development of blood vessel*	*62*	*1.1E-10*
**proliferation of cells**	**184**	**1.23E-10**
*vasculogenesis*	*57*	*1.59E-10*
*differentiation of connective tissue cells*	*47*	*2.99E-10*
*proliferation of tumor cell lines*	*95*	*4.31E-10*
*apoptosis of tumor cell lines*	*79*	*7.36E-10*
*development of cardiovascular system*	*70*	*4.06E-09*
*phosphorylation of protein*	*53*	*1.28E-08*
**necrosis**	**138**	**2.27E-08**
**cell death of tumor cell lines**	**90**	**2.47E-08**
*proliferation of connective tissue cells*	*45*	*5.19E-08*
*differentiation of adipocytes*	*22*	*5.46E-08*
*morphology of cells*	*87*	*7.31E-08*
*development of epithelial tissue*	*39*	*8.35E-08*
*migration of tumor cell lines*	*45*	*1.46E-07*
*neoplasia of tumor cell lines*	*23*	*2.86E-07*
*cell movement of tumor cell lines*	*52*	*3.23E-07*
*synthesis of DNA*	*34*	*3.74E-07*
*proliferation of muscle cells*	*30*	*4.05E-07*
*proliferation of smooth muscle cells*	*25*	*4.1E-07*
*blood pressure*	*27*	*6.81E-07*
*development of connective tissue*	*25*	*6.82E-07*
*migration of cells*	*116*	*2.76E-12*
*cell movement*	*124*	*8.07E-12*

**Table 8:** Top 25 biological processes associated with ZIPK knockdown (of 292 with *p* < 0.02).

Biological Process	DEGs	*p* value
*cell viability of gastrointestinal stromal tumor cell lines*	*6*	*2.09E-07*
*interphase of bone cancer cell lines*	*8*	*9.53E-06*
**necrosis**	**94**	**1.91E-05**
*DNA replication*	*17*	*2.52E-05*
**cell death of tumor cell lines**	**60**	**3.18E-05**
*metabolism of DNA*	*23*	*4.43E-05*
*cell viability*	*52*	*6.54E-05*
*mass of epigonadal fat pad*	*6*	*0.000107*
*G1 phase of bone cancer cell lines*	*6*	*0.000123*
*cell death of cerebral cortex cells*	*14*	*0.000209*
*checkpoint control*	*9*	*0.000222*
*S phase of tumor cell lines*	*9*	*0.000273*
*interphase*	*30*	*0.000313*
**proliferation of cells**	**114**	**0.000339**
*response of tumor cell lines*	*10*	*0.00036*
*arrest in cell cycle progression of epithelial cell lines*	*3*	*0.000367*
*endoreduplication of megakaryocytes*	*2*	*0.000369*
*replication of leukemia cell lines*	*2*	*0.000369*
*entry into cell cycle progression of fibroblasts*	*2*	*0.000369*
*proliferation of mesenchymal stem cells*	*5*	*0.000389*
**apoptosis**	**90**	**0.000457**
*cell death of central nervous system cells*	*16*	*0.000495*
*cell death of brain*	*16*	*0.000548*
*cell death of cortical neurons*	*11*	*0.000616*
*replication of centriole*	*4*	*0.00062*
*cell viability of gastrointestinal stromal tumor cell lines*	*6*	*2.09E-07*
*interphase of bone cancer cell lines*	*8*	*9.53E-06*

IPA was also utilized to identify DEGs that connect to other DEGs in biological processes or pathways. The top 10 DEGs with the most connections in the ROCK1- and ZIPK-knockdown datasets are listed in Table 9. Among these genes, HDAC1 was the only gene affected by knockdown of both ROCK1 and ZIPK, and was the top gene in the ZIPK group (with 14 neighbors) and fourth in the ROCK1-knockdown group (with 21 neighbors). MYC was the top gene in the ROCK1-knockdown group, which also contains three other transcription regulators (PPARG and VDR, in addition to HDAC1), all of which were up-regulated. Interestingly, up-regulated IL-6 is the number 2 gene in the ROCK1-knockdown group, and it has the most connections in this group at 28.

**Table 9:** Top 10 DEGs with multiple connections.

	Gene	Neighbors	Genes
**ROCK1 knockdown**	MYC	29	IL6, CEP57, STAT1, PIM2, MYO9A, BTRC, FBXW7, CAMK2D, BCR, ANGPTL4, STK38, IQGAP3, VDR, RAD21, RPN1, PIM1, PLOD3, CHCHD3, IGF2BP2, CDC25A, SQRDL, TAF12, CYR61, KDM5A, ADM, SNW1, CDKN2B, HDAC1, NEK11
	IL6	28	ABCG2, CCL11, SOCS3, FCGR1A, STAT1, PIM2, F2RL1, PTGS2, RARRES3, FCGR1B, CEBPD, VDR, BMP6, PTHLH, NNMT, MYC, PIM1, LBP, TNFSF13B, CDC25A, PTX3, IGFBP3, PPARG, GHR, IRF1, ADM, STX2, MAPK9
	STAT1	24	IL6, USP18, PARP9, SOCS3, IFNE, FCGR1A, TEK, TOM1L1, BTRC, PAWR, ANGPTL4, VDR, CCR1, SCGB3A2, MYC, LBP, STAT4, IGFBP3, AGTR1, PPARG, GHR, IRF1, IFNAR1, HDAC1
	HDAC1	21	PRKACA, NR1D2, CBX5, ESCO2, NUP54, STAT1, CREM, UHRF2, GMNN, UHRF1, CEBPD, NR4A2, MYC, CELF1, POLD3, KLF4, PPARG, BNIP3, TPD52L1, MAPK10, MAPK9
	PPARG	13	IL6, CCND3, THRB, STAT1, PTGS2, BCR, PTGES, PTHLH, KLF4, IGFBP3, TNFSF10, CCDC88A, HDAC1
	PRKACA	12	KCNJ2, CREM, MAP2, BTRC, PTGS2, PDE3A, UHRF1, PRKAR1A, CFL1, CCDC88A, KCNMA1, HDAC1
	MAPK9	9	IL6, MAP2, MAP3K2, MAP3K3, RARG, STAT4, PTPN22, UBE2N, HDAC1
	BTRC	9	PRKACA, STAT1, UBE2D1, UHRF1, MYC, CDC25A, DEPTOR, GHR, IFNAR1
	UBE2N	8	MID1, BIRC3, PC, RNF13, TOPORS, CELF1, PCCA, MAPK9
	VDR	8	IL6, CCND3, NR4A1, STAT1, MYC, IGFBP3, KDM5A, SNW1
**ZIPK knockdown**	HDAC1	14	CRYAB, PLK4, TOB1, CCNE1, BUB1B, LCOR, MAPK8, SATB1, NR1D2, ESCO2, NUP54, MCM6, RBL1, ZNF395
	MAPK8	11	TGFB3, TNFSF15, HIPK2, PLD1, DUSP4, APAF1, TLR4, PLAU, CDC25A, HDAC1, IL1R1
	VCAM1	9	IMMT, MCM5, MSI2, TIA1, MCM7, MLLT4, MCM3, HIST1H2BJ/HIST1H2BK, MCM6
	MCM3	9	MCM5, CCNE1, AGTR1, VCAM1, MCM10, CDC6, MCM7, MAPK6, MCM6
	MCM7	9	MCM5, CCNE1, TIPIN, VCAM1, MCM10, CDC6, MCM3, MCM6, RBL1
	CCNE1	9	SKP2, E2F5, CDC6, CDC25A, CDK6, HDAC1, MCM7, MCM3, RBL1
	MCM6	8	MCM5, ASF1B, TIPIN, VCAM1, MCM10, HDAC1, MCM7, MCM3
	RBL1	8	SKP2, CCNE1, CCND2, E2F5, CDK6, HDAC1, MCM7, MAPK6
	AGTR1	7	TRPV4, MYOCD, MLLT4, MCM3, INA, WEE1, HBEGF
	CDK6	6	CCNE1, CCND2, MCM10, CDC6, CDC25A, RBL1

In comparison to ROCK1 knockdown, all eight proteins encoded by the up-regulated DEGs in the ZIPK-knockdown group are localized to the nucleus and related to transcriptional regulation. Four of these eight genes belong to a nuclear enzyme family (minichromosome maintenance complex component: MCM 3, 6 and 7, together with CDK6) and are involved in control of the chromosomal replication pathway in the cell cycle. These genes are essential for eukaryotic DNA replication. This pathway was not observed in the ROCK1-knockdown group, which may provide another explanation for the decreased viability of ROCK1-knockdown SMCs (Fig. 5). This information is consistent with the pathway and biological function categories discussed above, and indicates again that both kinases are involved in regulation of the cell cycle and cell proliferation, but via different mechanisms. The majority of the DEGs (9 of 10) with the most connections were not shared by the two groups, indicating unique biological functions.

### Analysis of diseases associated with ROCK1 and ZIPK knockdown

In order to explore the pathological effects of ROCK1 and ZIPK expression in cells, DEGs were categorized in accordance with their association with different diseases. 127 different diseases were associated with DEGs in the ROCK1-knockdown group and 149 with the ZIPK-knockdown group. The top 25 pathological conditions from each group are presented in Tables 10 (ROCK1 knockdown) and 11 (ZIPK knockdown), based on their *p* values. Disease associations common to ROCK1 and ZIPK knockdown are depicted in bold in Tables 10 and 11. 18 of the 25 diseases in the ROCK1-knockdown group are cancer/tumor-related, 5 are immune- or autoimmune-related, and 2 are seizure-related. These observations are consistent with other ROCK1-knockdown microarray information, such as the higher up-regulation rate of 56% of affected genes in the cytokine sub-group, in contrast to only 36% up-regulation for all DEGs (Table 3). The higher protein levels of three pro-inflammatory cytokines (IL-6, IL-1β and MCP1 (CCL2)) in ROCK1-knockdown cells (Fig.s 3B and 4, and Table D in S1 File) provide further support for a role for ROCK1 in inflammation and immune-related processes. Almost all of the top 25 diseases in the ZIPK-knockdown group are cancer/tumor-related (the sole exception being mosaic variegated aneuploidy).

**Table 10:** Top 25 diseases associated with ROCK1 knockdown (FC ≥ 1.5).

Disease	DEGs	*p* value
**malignant neoplasm of abdomen**	**215**	**8.09E-09**
**metastasis**	**49**	**1.02E-08**
**epithelial neoplasia**	**261**	**3.31E-08**
**cancer**	**298**	**3.36E-08**
*arthritis*	*66*	*3.85E-08*
**solid tumor**	**255**	**6.59E-08**
**carcinoma**	**253**	**6.96E-08**
*growth of tumor*	*35*	*1.13E-07*
*neoplasia of tumor cell lines*	*23*	*2.86E-07*
*osteoarthritis*	*18*	*2.87E-07*
*seizures*	*34*	*3.4E-07*
*neoplasia of cells*	*29*	*1.47E-06*
*psoriasis*	*42*	*1.81E-06*
**metastatic colorectal cancer**	**19**	**1.95E-06**
*gastrointestinal tumor*	*169*	*2.01E-06*
*experimental autoimmune encephalomyelitis*	*22*	*2.01E-06*
*colorectal tumor*	*161*	*2.32E-06*
*uterine tumor*	*58*	*2.57E-06*
**digestive organ tumor**	**180**	**2.79E-06**
*metastasis of lung*	*10*	*2.8E-06*
*benign neoplasia*	*48*	*3.05E-06*
**colorectal cancer**	**160**	**3.06E-06**
**gastrointestinal tract cancer**	**167**	**3.44E-06**
*seizure disorder*	*35*	*3.52E-06*
*infection by Influenza A virus*	*7*	*9.86E-06*

**Table 11:** Top 25 diseases associated with ZIPK knockdown (FC ≥ 1.5).

Disease	DEGs	*p* value
**solid tumor**	**206**	**1.69E-13**
**carcinoma**	**204**	**2.93E-13**
**epithelial neoplasia**	**205**	**5.15E-12**
**cancer**	**228**	**4.2E-11**
**malignant neoplasm of abdomen**	**164**	**6.09E-10**
*adenocarcinoma*	*151*	*4.21E-08*
**colorectal cancer**	**123**	**3.46E-07**
**gastrointestinal tract cancer**	**127**	**7.23E-07**
**digestive organ tumor**	**134**	**2.28E-06**
*genital tumor*	*54*	*4.07E-06*
**metastatic colorectal cancer**	**15**	**6.54E-06**
*colon cancer*	*103*	*4.23E-05*
*colorectal carcinoma*	*104*	*4.74E-05*
*gastrointestinal carcinoma*	*105*	*7.01E-05*
**metastasis**	**30**	**0.000111**
*gastrointestinal adenocarcinoma*	*93*	*0.000178*
*colon carcinoma*	*94*	*0.000188*
*endometrial cancer*	*20*	*0.00022*
*prostatic tumor*	*30*	*0.000287*
*endometrial carcinoma*	*28*	*0.000326*
*mosaic variegated aneuploidy*	*2*	*0.000369*
*development of adenocarcinoma*	*4*	*0.000414*
*colon adenocarcinoma*	*90*	*0.000434*
*prostate cancer*	*29*	*0.000479*
*uterine serous papillary cancer*	*14*	*0.000719*

Finally, we investigated the effect of ROCK1 and ZIPK knockdown on phosphorylation of a well-known substrate, LC_20_ [19,28]. The level of expression of LC_20_ was unaffected by knockdown of either ROCK1 or ZIPK: LC_20_ was quantified in UASMC transfected with scrambled siRNA, ROCK1- or ZIPK-siRNA by western blotting with GAPDH as loading control. The levels of expression of LC_20_ in ZIPK- and ROCK1-knockdown cells relative to control cells were 95.1 ± 2.8% and 97.6 ± 3.0%, respectively (*n* = 14). Phosphorylation of LC_20_ at Thr18 and Ser19 [19,28] was then examined by western blotting with a phosphospecific antibody that recognizes LC_20_ only when phosphorylated at both sites. Knockdown of ROCK1 or ZIPK significantly reduced LC_20_ phosphorylation (Table 12 and Fig. B in S1 File).

**Table 12:** Effect of ROCK1 and ZIPK knockdown on myosin phosphorylation.

Cells	2P-LC_20_
Control	1.31 ± 0.09
ROCK1 knockdown	1.00 ± 0.07**
ZIPK knockdown	0.88 ± 0.07*

UASMC were transfected with siRNAs targeting ROCK1 or ZIPK or with negative control siRNA as described in the Materials and Methods section. Cells were lysed in SDS-gel sample buffer and subjected to SDS-PAGE and western blotting with anti-2P-LC_20_ (antibody recognizing LC_20_ only when phosphorylated at Thr18 and Ser19). 2P-LC_20_ levels are expressed relative to GAPDH as loading control. Values indicate the mean ± SEM (*n* = 20). *^,^**significantly different from control (*p < 0.001; **p < 0.01). Representative western blots are shown in Fig. B in S1 File.

## Discussion

Given that ROCK1 and ZIPK mediate important cellular functions and have been implicated in many pathological conditions, mainly vascular diseases such as hypertension, stroke, vasospasm, atherosclerosis, heart failure and pulmonary hypertension, as well as cancer, they are potentially useful therapeutic targets. Many compounds have been developed as inhibitors of ROCK or ZIPK and have been proposed to have therapeutic benefits for multiple diseases [10,49]. However, these compounds usually lack specificity because the ATP-binding sites of protein kinases, with which most chemical inhibitors interact, are quite conserved, which limits their therapeutic promise and usefulness as tools to investigate signaling pathways involving these kinases. As an alternative strategy, RNA interference (RNAi) is being widely utilized to deplete specific proteins from cells or tissues.

Targeting of gene transcripts using RNAi also provides a powerful approach to the analysis of gene function and to assess the role of specific proteins in particular cell functions. The use of microarray analysis to study gene expression patterns and profile changes is a powerful approach to identification of the biological functions of specific gene products. In this study, we have utilized RNAi and microarray analysis to further understand ROCK1 and ZIPK functions. This is the first attempt of its kind aimed at studying global gene expression changes resulting from kinase knockdown in order to identify unique genes regulated by ROCK1 and ZIPK, to explore novel functions of the two kinases and to test the hypothesis that ROCK1 commonly lies upstream of ZIPK. In this manuscript, we present the significant gene expression pattern variations and the analysis of the most relevant biological pathways and processes in ROCK1- and ZIPK-knockdown SMC to understand their potential functions. We confirmed 8 of the up- or down-regulated genes using qPCR, western blotting and/or ELISA: IL-1β and IL-6 (Figs. 3 and 4), MCP1 (CCL2) (Table D in S1 File), COX2, vEGF, ICAM, SOCS3 and ILK (data not shown). Our gene expression analysis clearly distinguished the effects of ROCK1 from ZIPK knockdown in CASMC. Numerous genes showed altered expression following kinase knockdown and several classes of gene functions were identified. It is interesting to note that both ROCK1 and ZIPK are involved in regulation of the cell cycle. Cell cycle: G1/S Checkpoint regulation was the principal pathway (based on the *p* value) affected by both knockdowns. However, the functional implications of the significant DEGs included in the pathway are unclear. As verified by the MTT test, ZIPK knockdown did not affect cell viability, whereas ROCK1 knockdown inhibited cell viability significantly. Gene expression changes may be due to a direct effect on transcriptional regulation or an indirect effect on the cytoskeleton. We did not observe any cell shape change by light microscopy in either ZIPK- or ROCK1-knockdown SMC. Furthermore, staining of the cytoskeleton with Alexa Fluor 388 phalloidin in kinase-knockdown cells did not reveal any effect on the structure of the actin cytoskeleton (data not shown), suggesting direct effects of ZIPK and ROCK1 knockdown on transcriptional regulation.

Accumulating evidence suggests that ROCK1 plays an important role in the regulation of cell proliferation and survival. ROCK is activated during the cell cycle at both the G1/S-phase and during mitosis. Over-expression of constitutively-active ROCK1 increases cell proliferation [50]. In contrast, inhibition of ROCK activity delays cytokinesis [51]. The G1-phase cell cycle checkpoint is the critical juncture at which DNA integrity is assessed and determines whether a cell commits to the cell cycle or reverts to the quiescent G0-phase. ROCK expression in 3T3 fibroblasts stimulates their progression into S-phase [52] while inhibition of ROCK activity in gastric cells impairs the G1/S-phase transition [53]. This is also supported by studies of cardiomyocytes showing that ROCK inhibition reduces the expression of G1/S-phase transition-dependent proteins [54]. A recent report indicated that ROCK1 knockdown decreased PDGF-BB-generated vascular SMC proliferation by inhibiting the expression of proliferating cell nuclear antigen (PCNA) and cyclin D1 [48]. Indeed, in our study ROCK1 knockdown significantly decreased cell proliferation and the expression of both CCND2 and 3 (cyclin D2 and 3; FC for CCND2 is-1.47), although cyclin D1 was unchanged.

Over-expression of ZIPK causes cell death via both caspase-dependent apoptosis and caspase-independent autophagy [23,55,56]. Cells that over-express ZIPK typically show signs of rounding, membrane blebbing, DNA fragmentation and detachment from the extracellular matrix. We observed a high incidence of altered DEGs associated with the cell cycle and nuclear location in ZIPK-knockdown SMC. Further study of these DEGs associated with ZIPK-knockdown may provide valuable insights into the mechanisms of cell death induced by ZIPK over-expression.

Inflammation is involved in most chronic diseases and the inflammatory response is regulated by diverse cytokines, chemokines, growth factors and cell adhesion molecules. These mediators are produced by multiple cell types, including mucosal epithelium, smooth muscle cells, glial cells and various leukocytes. Smooth muscle cells act as both sources and targets of these mediators, establishing an intricate, dynamic balance that maintains the inflammatory response [57,58]. Intestinal inflammation in human and animals has been widely shown to be associated with changes in contractility [57,59] and IL-1β was found to inhibit both initial and sustained contraction and LC_20_ phosphorylation stimulated by acetylcholine in the presence of an M2 receptor antagonist [60]. Inflammation is observed to various degrees in a diverse group of vascular diseases, including atherosclerosis and myocardial infarction. IL-1β and IL-6 are the two major pro-inflammatory cytokines involved in regulation of the inflammatory process and exhibit increased levels in these pathological situations. Epidemiologically, vascular inflammation is detected by enhanced circulating IL-6 produced by activated inflammatory cells within the vessel wall. The immune response following inflammation directly induces apoptosis and indirectly increases cell death by signaling cellular death programs [61,62], although the precise mechanisms are unknown. Interestingly, in this study we observed that cytokine gene expression, specifically of IL-6, IL-1β and MCP1 (CCL2), was differently affected by ROCK1 and ZIPK knockdown in CASMC, i.e., ROCK1 knockdown increased while ZIPK knockdown decreased their expression. As noted earlier, ROCK1 knockdown by ~80% reduced ROCK2 protein expression by ~35%, making it difficult to distinguish between the effects of ROCK1 and ROCK2. However, knockdown of ROCK2 at the protein level by ~55% had no effect on IL-6 expression suggesting that ROCK1 is likely the isoform involved in regulation of IL-6 expression.

Although microarray analysis provides a detailed transcription profile, it has limitations associated with the data analysis. Some genes with higher variations (in both knockdown and microarray assays) are easily excluded by *p* values because of the low number of replicates (commonly, *n* = 3). IL-1β is a good example: its differential expression was verified by both western blotting at the protein level and by qRT-PCR at the mRNA level, but it was excluded from the array dataset because of a high *p* value. We used FC > 1.5 and *q* value < 0.05 as the criteria to identify DEGs, but some genes excluded by these criteria were in fact significantly altered by kinase knockdown. For example, IL-6 (FC = -1.26) and ROCK1 (FC = -1.006) were found to be down- and up-regulated, respectively, in ZIPK-knockdown cells at the protein level. RNAi also has its limitations, which must be taken into account when interpreting the effects of down-regulation of specific target genes by siRNA. For example, as shown in this study, the extents of down-regulation of ROCK1 (~80%) and ZIPK (~50%) at the protein level were quite different. ZIPK down-regulation affected ROCK1 expression, which was increased by ~30%, whereas ROCK1 down-regulation had no effect on ZIPK expression. Furthermore, ROCK1 down-regulation (by ~80%) reduced the level of ROCK2 expression (by ~35%).

LC_20_ has been identified as a substrate of both ROCK1 and ZIPK, at least *in vitro*, with phosphorylation occurring at Thr18 and Ser19 [19,28]. We showed that down-regulation of ROCK1 or ZIPK reduced LC_20_ phosphorylation (Table 12 and Fig. B in S1 File). This could be explained by direct phosphorylation of LC_20_ by both kinases or activation of ROCK1 leading to activation of ZIPK, which phosphorylates LC_20_.

In summary, this study has identified sets of genes whose expression is altered by knockdown of ROCK1 or ZIPK in vascular SMC, as well as signaling pathways, biological processes and diseases involving each of these kinases. At this point we cannot conclude whether these gene expression changes represent global effects of ROCK1 and ZIPK knockdown caused by key upstream regulators, or if they are genes that are directly targeted by the respective kinases. In addition, certain compensatory changes may occur over time, as opposed to primary effects of gene knockdown. Further studies focused on these genes may provide valuable insights into the physiological and pathophysiological roles of ROCK1 and ZIPK in vascular SMC. For example, the opposing effects of ROCK1 and ZIPK knockdown on the expression of IL-6, IL-1β and MCP1 (CCL2) suggest that ZIPK may be a more suitable therapeutic target in the case of chronic inflammatory diseases. Finally, the fact that, while knockdown of ROCK1 or ZIPK has a number of similar effects, the differences are far greater, suggesting that ROCK1-mediated activation of ZIPK may be involved in only a subset of signaling pathways involving these kinases.

## Supporting Information

S1 File Fig. A, Quantification of ZIPK mRNA levels in CASMC transfected with ZIPK or ROCK1 siRNAs. CASMCs were transfected with siRNA to ZIPK or ROCK1 or with negative control siRNA. Cells were lysed 48 h later for qRT-PCR to quantify ZIPK mRNA levels. Values represent means ± SEM (*n* = 9). *significantly different from control (*p* < 0.001). **Fig. B, Effect of ROCK1 and ZIPK knockdown on myosin phosphorylation**. Representative western blots of control, ZIPK- and ROCK1-knockdown UASMC with anti-2P-LC_20_ (antibody recognizing LC_20_ only when phosphorylated at Thr18 and Ser19) and GAPDH as loading control. See Table 12 for cumulative quantitative data. **Table A, ROCK1 and ZIPK knockdown in UASMC at the protein level**. UASMC were transfected with siRNAs targeting ROCK1 or ZIPK or with negative control siRNA as described in the Materials and Methods section. The efficiency of knockdown at the protein level was determined by western blotting. ROCK1 and ZIPK signals were normalized to GAPDH. Values are expressed relative to levels in control cells (means ± SD, *n* = 11 for ROCK1 knockdown and *n* = 8 for ZIPK knockdown). *significantly different from control (*p* < 0.001). **Table B, Genes whose expression is altered by ROCK1 knockdown. Table C, Genes whose expression is altered by ZIPK knockdown. Table D, Effects of ZIPK and ROCK1 knockdown on cytokine secretion**. A Human Custom Multi-Analyte ELISArray kit (CELISA-CMEH0590A) was purchased from Qiagen. The indicated cytokines were assayed in the medium of CASMC transfected with control siRNA (Control) and CASMC transfected with siRNA to ZIPK (ZIPK knockdown) or ROCK1 (ROCK1 knockdown). Positive controls provided with the kit verified the viability of the assay for each cytokine. Negative controls indicated that, of the 6 cytokines listed, IL-1α, MCP1 and GROα were secreted at detectable levels. Values indicate absorbance at 450 nm ± S.E.M. (*n* = 4). *p < 0.05 compared to Control.
(PDF)
