# Supplementary material for: The Effects of Knockdown of Rho-Associated Kinase 1 and Zipper-Interacting Protein Kinase on Gene Expression and Function in Cultured Human Arterial Smooth Muscle Cells
Source: PLoS One. 2015 Feb 27;10(2):e0116969. doi: 10.1371/journal.pone.0116969 (PMC4344299; doi:10.1371/journal.pone.0116969)
Supplement: S1 File — CASMCs were transfected with siRNA to ZIPK or ROCK1 or with negative control siRNA. Cells were lysed 48 h later for qRT-PCR to quantify ZIPK mRNA levels. Values represent means ± SEM (n = 9). *significantly different from control (p < 0.001). Fig. B, Effect of ROCK1 and ZIPK knockdown on myosin phosphorylation. Representative western blots of control, ZIPK- and ROCK1-knockdown UASMC with anti-2P-LC20 (antibody recognizing LC20 only when phosphorylated at Thr18 and Ser19) and GAPDH as loading control. See Table 12 for cumulative quantitative data. Table A, ROCK1 and ZIPK knockdown in UASMC at the protein level. UASMC were transfected with siRNAs targeting ROCK1 or ZIPK or with negative control siRNA as described in the Materials and Methods section. The efficiency of knockdown at the protein level was determined by western blotting. ROCK1 and ZIPK signals were normalized to GAPDH. Values are expressed relative to levels in control cells (means ± SD, n = 11 for ROCK1 knockdown and n = 8 for ZIPK knockdown). *significantly different from control (p < 0.001). Table B, Genes whose expression is altered by ROCK1 knockdown. Table C, Genes whose expression is altered by ZIPK knockdown. Table D, Effects of ZIPK and ROCK1 knockdown on cytokine secretion. A Human Custom Multi-Analyte ELISArray kit (CELISA-CMEH0590A) was purchased from Qiagen. The indicated cytokines were assayed in the medium of CASMC transfected with control siRNA (Control) and CASMC transfected with siRNA to ZIPK (ZIPK knockdown) or ROCK1 (ROCK1 knockdown). Positive controls provided with the kit verified the viability of the assay for each cytokine. Negative controls indicated that, of the 6 cytokines listed, IL-1α, MCP1 and GROα were secreted at detectable levels. Values indicate absorbance at 450 nm ± S.E.M. (n = 4). *p < 0.05 compared to Control. (PDF) [file pone.0116969.s001.pdf]

Supporting Information

Figure A. Quantification of ZIPK mRNA levels in CASMC transfected with ZIPK or ROCK1 siRNAs. CASMCs were transfected with siRNA to ZIPK or ROCK1 or with negative control siRNA. Cells were lysed 48 h later for qRT-PCR to quantify ZIPK mRNA levels. Values represent means ± SEM ($n = 9$). *significantly different from control ($p < 0.001$).

Figure B. Effect of ROCK1 and ZIPK knockdown on myosin phosphorylation. Representative western blots of control, ZIPK- and ROCK1-knockdown UASMC with anti-2P-LC_{20} (antibody recognizing LC_{20} only when phosphorylated at Thr18 and Ser19) and GAPDH as loading control. See Table 12 for cumulative quantitative data.

Table A. ROCK1 and ZIPK knockdown in UASMC at the protein level.

Table B. Genes whose expression is altered by ROCK1 knockdown.

Table C. Genes whose expression is altered by ZIPK knockdown.

Table D. Effects of ZIPK and ROCK1 knockdown on cytokine secretion.

Figure A

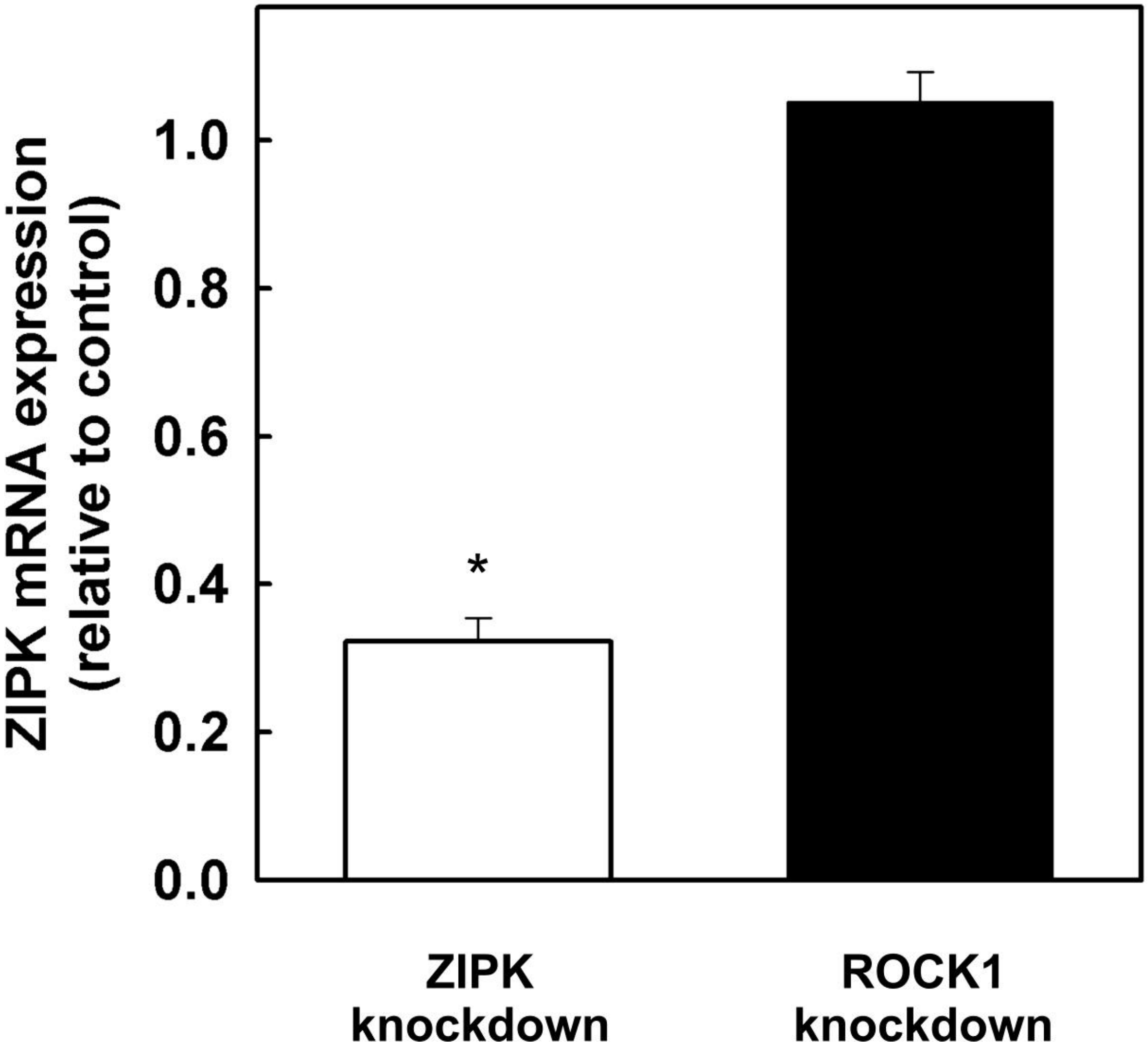

ZIPK mRNA expression
(relative to control)
0.0
0.2
0.4
0.6
0.8
1.0
*
ZIPK
knockdown
ROCK1
knockdown

Figure B

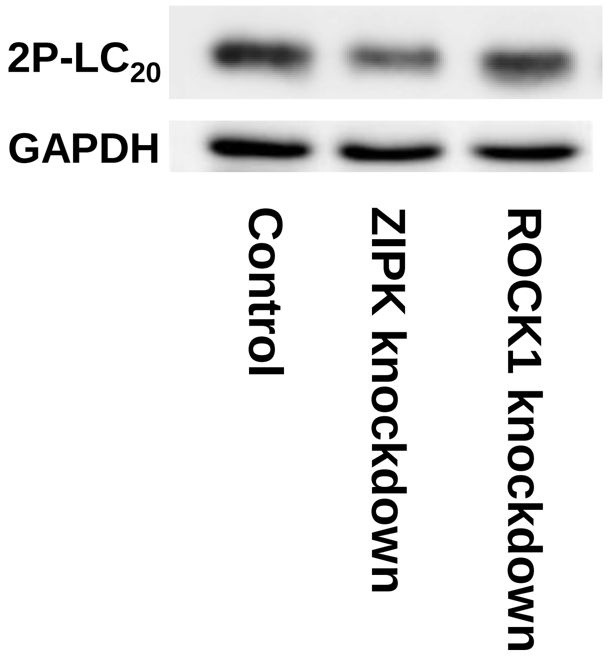

Table A. ROCK1 and ZIPK knockdown in UASMC at the protein level.

Kinase	ROCK1 knockdown	ZIPK knockdown
ROCK1	0.22 ± 0.12*	1.33 ± 0.15*
ZIPK	0.93 ± 0.25	0.52 ± 0.12*

UASMC were transfected with siRNAs targeting ROCK1 or ZIPK or with negative control siRNA as described in the Materials and Methods section. The efficiency of knockdown at the protein level was determined by western blotting. ROCK1 and ZIPK signals were normalized to GAPDH. Values are expressed relative to levels in control cells (means ± SD, $n = 11$ for ROCK1 knockdown and $n = 8$ for ZIPK knockdown). *significantly different from control ($p < 0.001$).

Table B. Genes whose expression is altered by ROCK1 knockdown.

Gene Symbol: Gene Name	Fold Change
LMLN: leishmanolysin-like (metallopeptidase M8 family)	6.432
GDF5: growth differentiation factor 5	3.648
LBP: lipopolysaccharide binding protein	3.598
GCNT4: glucosaminyl (N-acetyl) transferase 4, core 2	2.841
PNPLA3: patatin-like phospholipase domain containing 3	2.825
VDR: vitamin D (1,25- dihydroxyvitamin D3) receptor	2.632
OLFML2B: olfactomedin-like 2B	2.624
ALPK2: alpha-kinase 2	2.597
C1orf110: chromosome 1 open reading frame 110	2.595
ANGPTL4: angiopoietin-like 4	2.536
SLC1A3: solute carrier family 1 (glial high affinity glutamate transporter), member 3	2.521
HDAC1: histone deacetylase 1	2.514
CCL7: chemokine (C-C motif) ligand 7	2.344
ZC3HAV1L: zinc finger CCCH-type, antiviral 1-like	2.304
SQRDL: sulfide quinone reductase-like (yeast)	2.278
IL6: interleukin 6 (interferon, beta 2)	2.263
SPATA17: spermatogenesis associated 17	2.221
C1orf198: chromosome 1 open reading frame 198	2.207
SAMD15: sterile alpha motif domain containing 15	2.204
FAM49B: family with sequence similarity 49, member B	2.203
VGLL3: vestigial like 3 (Drosophila)	2.197
EIF5A2: eukaryotic translation initiation factor 5A2	2.193
SOCS3: suppressor of cytokine signaling 3	2.153
AOX1: aldehyde oxidase 1	2.14
KIAA1467: KIAA1467	2.133
UBE2D1: ubiquitin-conjugating enzyme E2D 1	2.117
DDAH1: dimethylarginine dimethylaminohydrolase 1	2.112
EFHC2: EF-hand domain (C-terminal) containing 2	2.101
TNFRSF11B: tumor necrosis factor receptor superfamily, member 11b	2.083
C7orf63: chromosome 7 open reading frame 63	2.064
ROPN1L: rhophilin associated tail protein 1-like	2.027
RNF144B: ring finger protein 144B	2.02
NDUFA2: NADH dehydrogenase (ubiquinone) 1 alpha subcomplex, 2, 8kDa	2.006
C6orf165: chromosome 6 open reading frame 165	1.994
CARS2: cysteinyl-tRNA synthetase 2, mitochondrial (putative)	1.983
CCDC176: coiled-coil domain containing 176	1.98
ADAMTS15: ADAM metallopeptidase with thrombospondin type 1 motif, 15	1.976
TPD52L1: tumor protein D52-like 1	1.964
HIST1H2BJ/HIST1H2BK: histone cluster 1, H2bk	1.963

MID1: midline 1 (Opitz/BBB syndrome)	1.961
FBXO16: F-box protein 16	1.953
COL15A1: collagen, type XV, alpha 1	1.916
AP3M2: adaptor-related protein complex 3, mu 2 subunit	1.912
SERPINB9: serpin peptidase inhibitor, clade B (ovalbumin), member 9	1.893
GALNT15: UDP-N-acetyl-alpha-D-galactosamine:polypeptide N-acetylgalactosaminyltransferase 15	1.891
ARMC9: armadillo repeat containing 9	1.874
mir-145: microRNA 145	1.865
LRRC6: leucine rich repeat containing 6	1.86
CPA4: carboxypeptidase A4	1.856
SPIN4: spindlin family, member 4	1.855
LMCD1: LIM and cysteine-rich domains 1	1.849
VWA9: von Willebrand factor A domain containing 9	1.832
NPR3: natriuretic peptide receptor C/guanylate cyclase C (atrionatriuretic peptide receptor C)	1.83
NNMT: nicotinamide N-methyltransferase	1.826
TMEM144: transmembrane protein 144	1.822
ENTPD7: ectonucleoside triphosphate diphosphohydrolase 7	1.822
NTF3: neurotrophin 3	1.815
UGCG: UDP-glucose ceramide glucosyltransferase	1.811
PKNOX2: PBX/knotted 1 homeobox 2	1.804
PIH1D2: PIH1 domain containing 2	1.801
HSD11B1: hydroxysteroid (11-beta) dehydrogenase 1	1.792
MEST: mesoderm specific transcript	1.786
PPIL6: peptidylprolyl isomerase (cyclophilin)-like 6	1.786
STAT4: signal transducer and activator of transcription 4	1.775
DPH2: DPH2 homolog (S. cerevisiae)	1.765
ARL13B: ADP-ribosylation factor-like 13B	1.764
DEPTOR: DEP domain containing MTOR-interacting protein	1.759
ADA: adenosine deaminase	1.758
mir-622: microRNA 622	1.751
POLR3D: polymerase (RNA) III (DNA directed) polypeptide D, 44kDa	1.745
EML4: echinoderm microtubule associated protein like 4	1.744
STX2: syntaxin 2	1.742
OSCP1: organic solute carrier partner 1	1.737
SYTL2: synaptotagmin-like 2	1.729
TNFSF10: tumor necrosis factor (ligand) superfamily, member 10	1.729
SHANK2: SH3 and multiple ankyrin repeat domains 2	1.728
KCNK15: potassium channel, subfamily K, member 15	1.724
GDF5OS: growth differentiation factor 5 opposite strand	1.722
C9orf116: chromosome 9 open reading frame 116	1.722
POLD3: polymerase (DNA-directed), delta 3, accessory subunit	1.72
SORBS2: sorbin and SH3 domain containing 2	1.72

IQCK: IQ motif containing K	1.715
CDC42EP3: CDC42 effector protein (Rho GTPase binding) 3	1.712
RARRES3: retinoic acid receptor responder (tazarotene induced) 3	1.711
CCDC113: coiled-coil domain containing 113	1.71
KCNJ6: potassium inwardly-rectifying channel, subfamily J, member 6	1.696
FAM87B: family with sequence similarity 87, member B	1.692
WDR65: WD repeat domain 65	1.691
MOK: MOK protein kinase	1.688
STK3: serine/threonine kinase 3	1.684
TRIB2: tribbles homolog 2 (Drosophila)	1.684
CHCHD3: coiled-coil-helix-coiled-coil-helix domain containing 3	1.683
UHRF1: ubiquitin-like with PHD and ring finger domains 1	1.677
MICB: MHC class I polypeptide-related sequence B	1.674
NUP54: nucleoporin 54kDa	1.662
MT1F: metallothionein 1F	1.66
CCNE2: cyclin E2	1.659
ANKEF1: ankyrin repeat and EF-hand domain containing 1	1.653
KCNJ2: potassium inwardly-rectifying channel, subfamily J, member 2	1.652
GNL3L: guanine nucleotide binding protein-like 3 (nucleolar)-like	1.65
CDC25A: cell division cycle 25A	1.65
CCNJ: cyclin J	1.648
MT1X: metallothionein 1X	1.643
IFI35: interferon-induced protein 35	1.643
ZNF100: zinc finger protein 100	1.643
FBXO36: F-box protein 36	1.643
FJX1: four jointed box 1 (Drosophila)	1.64
CPSF4: cleavage and polyadenylation specific factor 4, 30kDa	1.639
RBM24: RNA binding motif protein 24	1.636
NUAK2: NUAK family, SNF1-like kinase, 2	1.633
ESCO2: establishment of sister chromatid cohesion N-acetyltransferase 2	1.629
NEK11: NIMA-related kinase 11	1.627
STAT1: signal transducer and activator of transcription 1, 91kDa	1.627
PIM1: pim-1 oncogene	1.625
NEXN: nexilin (F actin binding protein)	1.625
STEAP1: six transmembrane epithelial antigen of the prostate 1	1.625
BLZF1: basic leucine zipper nuclear factor 1	1.621
C17orf97: chromosome 17 open reading frame 97	1.621
MNS1: meiosis-specific nuclear structural 1	1.616
RSPH4A: radial spoke head 4 homolog A (Chlamydomonas)	1.616
DNAH7: dynein, axonemal, heavy chain 7	1.615
SDC4: syndecan 4	1.614
C11orf70: chromosome 11 open reading frame 70	1.613
NME5: NME/NM23 family member 5	1.613

TIPIN: TIMELESS interacting protein	1.599
MAS1: MAS1 oncogene	1.598
IFITM1: interferon induced transmembrane protein 1	1.594
FAM198B: family with sequence similarity 198, member B	1.592
CYR61: cysteine-rich, angiogenic inducer, 61	1.59
CEBPD: CCAAT/enhancer binding protein (C/EBP), delta	1.586
CTGF: connective tissue growth factor	1.58
PARP9: poly (ADP-ribose) polymerase family, member 9	1.577
MTF1: metal-regulatory transcription factor 1	1.576
FSIP1: fibrous sheath interacting protein 1	1.576
FAM43A: family with sequence similarity 43, member A	1.575
PPIF: peptidylprolyl isomerase F	1.574
IGF2BP2: insulin-like growth factor 2 mRNA binding protein 2	1.573
RAB36: RAB36, member RAS oncogene family	1.573
PAWR: PRKC, apoptosis, WT1, regulator	1.572
CELF2: CUGBP, Elav-like family member 2	1.571
POLR1C: polymerase (RNA) I polypeptide C, 30kDa	1.57
C20orf96: chromosome 20 open reading frame 96	1.568
TEX9: testis expressed 9	1.565
PRPS1: phosphoribosyl pyrophosphate synthetase 1	1.564
BNIP3: BCL2/adenovirus E1B 19kDa interacting protein 3	1.562
SDCCAG3: serologically defined colon cancer antigen 3	1.562
BBS5: Bardet-Biedl syndrome 5	1.561
BTN3A1: butyrophilin, subfamily 3, member A1	1.56
AGK: acylglycerol kinase	1.56
mir-199: microRNA 199a-2	1.56
ANKRD13A: ankyrin repeat domain 13A	1.56
PRUNE: prune exopolyphosphatase	1.559
PIM2: pim-2 oncogene	1.555
CCL8: chemokine (C-C motif) ligand 8	1.553
SRPR: signal recognition particle receptor (docking protein)	1.553
EFHC1: EF-hand domain (C-terminal) containing 1	1.548
FANK1: fibronectin type III and ankyrin repeat domains 1	1.546
DDIT4L: DNA-damage-inducible transcript 4-like	1.546
BPNT1: 3'(2'), 5'-bisphosphate nucleotidase 1	1.546
CEP57: centrosomal protein 57kDa	1.545
ADM: adrenomedullin	1.544
RPSAP52: ribosomal protein SA pseudogene 52	1.544
SLC2A1: solute carrier family 2 (facilitated glucose transporter), member 1	1.544
BMP2K: BMP2 inducible kinase	1.542
KIAA1737: KIAA1737	1.541
NFIL3: nuclear factor, interleukin 3 regulated	1.54
OFD1: oral-facial-digital syndrome 1	1.54

WDR4: WD repeat domain 4	1.539
ZNF528: zinc finger protein 528	1.538
CLMN: calmin (calponin-like, transmembrane)	1.535
WDR12: WD repeat domain 12	1.533
RHOBTB3: Rho-related BTB domain containing 3	1.533
SOD2: superoxide dismutase 2, mitochondrial	1.531
XAGE-4: XAGE-4 protein	1.531
ANK3: ankyrin 3, node of Ranvier (ankyrin G)	1.529
DCDC1: doublecortin domain containing 1	1.527
CCL11: chemokine (C-C motif) ligand 11	1.527
IRF1: interferon regulatory factor 1	1.527
EXOSC2: exosome component 2	1.525
LIMA1: LIM domain and actin binding 1	1.525
PSME2: proteasome (prosome, macropain) activator subunit 2 (PA28 beta)	1.523
PTX3: pentraxin 3, long	1.523
LACTB2: lactamase, beta 2	1.523
USP18: ubiquitin specific peptidase 18	1.521
SNORD76: small nucleolar RNA, C/D box 76	1.521
GPC4: glypican 4	1.52
SHQ1: SHQ1, H/ACA ribonucleoprotein assembly factor	1.518
EBF2: early B-cell factor 2	1.518
FAM227A: family with sequence similarity 227, member A	1.517
ENTPD3: ectonucleoside triphosphate diphosphohydrolase 3	1.517
CCR1: chemokine (C-C motif) receptor 1	1.516
PAXIP1: PAX interacting (with transcription-activation domain) protein 1	1.51
MYC: v-myc myelocytomatosis viral oncogene homolog (avian)	1.508
SNORD78: small nucleolar RNA, C/D box 78	1.507
AADAT: aminoadipate aminotransferase	1.507
HIST1H2BM: histone cluster 1, H2bm	1.503
DHRS3: dehydrogenase/reductase (SDR family) member 3	1.502
C19orf40: chromosome 19 open reading frame 40	1.501
ARMCX1: armadillo repeat containing, X-linked 1	-1.5
ITGA10: integrin, alpha 10	-1.5
EBF3: early B-cell factor 3	-1.502
FAM102B: family with sequence similarity 102, member B	-1.503
SFT2D1: SFT2 domain containing 1	-1.504
CDKN2B: cyclin-dependent kinase inhibitor 2B (p15, inhibits CDK4)	-1.504
PHLDB1: pleckstrin homology-like domain, family B, member 1	-1.507
GPR4: G protein-coupled receptor 4	-1.508
TRO: trophinin	-1.508
CREM: cAMP responsive element modulator	-1.511
FBXW7: F-box and WD repeat domain containing 7, E3 ubiquitin protein ligase	-1.513
HENMT1: HEN1 methyltransferase homolog 1 (Arabidopsis)	-1.514

CRABP2: cellular retinoic acid binding protein 2	-1.514
B4GALT3: UDP-Gal:betaGlcNAc beta 1,4- galactosyltransferase, polypeptide 3	-1.515
INPP4B: inositol polyphosphate-4-phosphatase, type II, 105kDa	-1.516
CHPF2: chondroitin polymerizing factor 2	-1.516
ZNF25: zinc finger protein 25	-1.517
KCNMA1: potassium large conductance calcium-activated channel, subfamily M, alpha member 1	-1.517
UHRF2: ubiquitin-like with PHD and ring finger domains 2, E3 ubiquitin protein ligase	-1.518
CD164: CD164 molecule, sialomucin	-1.518
CD93: CD93 molecule	-1.521
SERTAD4: SERTA domain containing 4	-1.522
KDELR1: KDEL (Lys-Asp-Glu-Leu) endoplasmic reticulum protein retention receptor 1	-1.522
PSKH1: protein serine kinase H1	-1.523
BZW1: basic leucine zipper and W2 domains 1	-1.524
CDKL4: cyclin-dependent kinase-like 4	-1.525
PLEKHH2: pleckstrin homology domain containing, family H (with MyTH4 domain) member 2	-1.526
PLCD1: phospholipase C, delta 1	-1.526
CCDC88A: coiled-coil domain containing 88A	-1.528
SLC5A3: solute carrier family 5 (sodium/myo-inositol cotransporter), member 3	-1.528
SLC33A1: solute carrier family 33 (acetyl-CoA transporter), member 1	-1.529
ABI3: ABI family, member 3	-1.53
FGF10: fibroblast growth factor 10	-1.53
PARM1: prostate androgen-regulated mucin-like protein 1	-1.531
PRPH: peripherin	-1.534
TNFSF13B: tumor necrosis factor (ligand) superfamily, member 13b	-1.534
ADAMTS1: ADAM metallopeptidase with thrombospondin type 1 motif, 1	-1.535
OSTN: osteocrin	-1.535
HACL1: 2-hydroxyacyl-CoA lyase 1	-1.536
EIF3J: eukaryotic translation initiation factor 3, subunit J	-1.537
F2RL1: coagulation factor II (thrombin) receptor-like 1	-1.537
CD274: CD274 molecule	-1.538
MAPK10: mitogen-activated protein kinase 10	-1.538
CAPN1: calpain 1, (mu/I) large subunit	-1.538
GAS2L3: growth arrest-specific 2 like 3	-1.538
SNW1: SNW domain containing 1	-1.539
TIMP4: TIMP metallopeptidase inhibitor 4	-1.54
TAS2R31: taste receptor, type 2, member 31	-1.541
SNX30: sorting nexin family member 30	-1.541
TMEM217: transmembrane protein 217	-1.542
FCGR1A: Fc fragment of IgG, high affinity Ia, receptor (CD64)	-1.542
PRSS35: protease, serine, 35	-1.543
PPP3R1: protein phosphatase 3, regulatory subunit B, alpha	-1.543
CYSLTR1: cysteinyl leukotriene receptor 1	-1.544

LPAR6: lysophosphatidic acid receptor 6	-1.545
KIAA1161: KIAA1161	-1.545
PRKAR1A: protein kinase, cAMP-dependent, regulatory, type I, alpha	-1.546
GALNT7: UDP-N-acetyl-alpha-D-galactosamine:polypeptide N-acetylgalactosaminyltransferase 7 (GalNAc-T7)	-1.548
GREM2: gremlin 2, DAN family BMP antagonist	-1.553
ELOVL5: ELOVL fatty acid elongase 5	-1.555
MASP1: mannan-binding lectin serine peptidase 1 (C4/C2 activating component of Ra-reactive factor)	-1.555
POC1B-GALNT4: POC1B-GALNT4 readthrough	-1.555
STK19: serine/threonine kinase 19	-1.556
RASA4CP: RAS p21 protein activator 4C, pseudogene	-1.557
DDAH2: dimethylarginine dimethylaminohydrolase 2	-1.557
MAP3K3: mitogen-activated protein kinase kinase kinase 3	-1.557
CSPG4: chondroitin sulfate proteoglycan 4	-1.557
ARHGAP12: Rho GTPase activating protein 12	-1.558
ABCG2: ATP-binding cassette, sub-family G (WHITE), member 2	-1.559
RAD51AP1: RAD51 associated protein 1	-1.559
AP1M1: adaptor-related protein complex 1, mu 1 subunit	-1.561
FCGR1B: Fc fragment of IgG, high affinity Ib, receptor (CD64)	-1.562
SLC35D2: solute carrier family 35, member D2	-1.562
ULBP2: UL16 binding protein 2	-1.562
DCUN1D1: DCN1, defective in cullin neddylation 1, domain containing 1	-1.564
GGA2: golgi-associated, gamma adaptin ear containing, ARF binding protein 2	-1.565
KDM5A: lysine (K)-specific demethylase 5A	-1.565
CYB5B: cytochrome b5 type B (outer mitochondrial membrane)	-1.569
TMEM140: transmembrane protein 140	-1.571
THRB: thyroid hormone receptor, beta	-1.571
CPEB2: cytoplasmic polyadenylation element binding protein 2	-1.572
PIGB: phosphatidylinositol glycan anchor biosynthesis, class B	-1.573
SHC4: SHC (Src homology 2 domain containing) family, member 4	-1.574
SGIP1: SH3-domain GRB2-like (endophilin) interacting protein 1	-1.576
ICAM2: intercellular adhesion molecule 2	-1.576
ITGA2: integrin, alpha 2 (CD49B, alpha 2 subunit of VLA-2 receptor)	-1.576
SDC3: syndecan 3	-1.577
ADAMTS5: ADAM metallopeptidase with thrombospondin type 1 motif, 5	-1.577
GM2A: GM2 ganglioside activator	-1.578
LRRFIP1: leucine rich repeat (in FLII) interacting protein 1	-1.578
FSTL3: follistatin-like 3 (secreted glycoprotein)	-1.579
MAP2: microtubule-associated protein 2	-1.58
CFL1: cofilin 1 (non-muscle)	-1.58
AS3MT: arsenic (+3 oxidation state) methyltransferase	-1.581
WNT16: wingless-type MMTV integration site family, member 16	-1.583
APH1B: APH1B gamma secretase subunit	-1.584

SHKBP1: SH3KBP1 binding protein 1	-1.584
MAPK9: mitogen-activated protein kinase 9	-1.585
CST2: cystatin SA	-1.586
GOLIM4: golgi integral membrane protein 4	-1.586
SH3PXD2B: SH3 and PX domains 2B	-1.587
PPME1: protein phosphatase methylesterase 1	-1.588
CBX5: chromobox homolog 5	-1.588
PTPN22: protein tyrosine phosphatase, non-receptor type 22 (lymphoid)	-1.593
FAM180A: family with sequence similarity 180, member A	-1.594
LPPR2: lipid phosphate phosphatase-related protein type 2	-1.599
FOXN2: forkhead box N2	-1.599
ELTD1: EGF, latrophilin and seven transmembrane domain containing 1	-1.601
AIP: aryl hydrocarbon receptor interacting protein	-1.601
BABAM1: BRISC and BRCA1 A complex member 1	-1.602
AKT1S1: AKT1 substrate 1 (proline-rich)	-1.605
ATP1B1: ATPase, Na+/K+ transporting, beta 1 polypeptide	-1.607
IQGAP3: IQ motif containing GTPase activating protein 3	-1.608
SNORD64: small nucleolar RNA, C/D box 64	-1.609
SCUBE3: signal peptide, CUB domain, EGF-like 3	-1.609
CAMK2D: calcium/calmodulin-dependent protein kinase II delta	-1.61
JAG1: jagged 1	-1.61
NTN4: netrin 4	-1.612
PTPRM: protein tyrosine phosphatase, receptor type, M	-1.615
THSD4: thrombospondin, type I, domain containing 4	-1.616
RAPGEF5: Rap guanine nucleotide exchange factor (GEF) 5	-1.618
RAB11FIP2: RAB11 family interacting protein 2 (class I)	-1.618
FLOT1: flotillin 1	-1.619
COMMD10: COMM domain containing 10	-1.623
MORN4: MORN repeat containing 4	-1.623
ATE1: arginyltransferase 1	-1.625
PAN2: PAN2 poly(A) specific ribonuclease subunit homolog (S. cerevisiae)	-1.626
TOM1L1: target of myb1 (chicken)-like 1	-1.628
MN1: meningioma (disrupted in balanced translocation) 1	-1.629
FRY: furry homolog (Drosophila)	-1.631
ASPM: asp (abnormal spindle) homolog, microcephaly associated (Drosophila)	-1.631
MICU1: mitochondrial calcium uptake 1	-1.633
GPRC5A: G protein-coupled receptor, family C, group 5, member A	-1.634
VEGFC: vascular endothelial growth factor C	-1.638
FBXO27: F-box protein 27	-1.643
LAMTOR3: late endosomal/lysosomal adaptor, MAPK and MTOR activator 3	-1.643
PROSC: proline synthetase co-transcribed homolog (bacterial)	-1.645
BCRP2: breakpoint cluster region pseudogene 2	-1.65
SDPR: serum deprivation response	-1.651

USP5: ubiquitin specific peptidase 5 (isopeptidase T)	-1.654
C20orf194: chromosome 20 open reading frame 194	-1.661
AGTR1: angiotensin II receptor, type 1	-1.664
CCDC28A: coiled-coil domain containing 28A	-1.664
ZDHHC18: zinc finger, DHHC-type containing 18	-1.665
STX5: syntaxin 5	-1.665
B4GALT1: UDP-Gal:betaGlcNAc beta 1,4- galactosyltransferase, polypeptide 1	-1.666
CMTM3: CKLF-like MARVEL transmembrane domain containing 3	-1.67
APOLD1: apolipoprotein L domain containing 1	-1.67
TSPAN4: tetraspanin 4	-1.67
PCCA: propionyl CoA carboxylase, alpha polypeptide	-1.674
CRAT: carnitine O-acetyltransferase	-1.674
DENND6A: DENN/MADD domain containing 6A	-1.675
LYPLAL1: lysophospholipase-like 1	-1.676
VLDLR: very low density lipoprotein receptor	-1.678
LNPEP: leucyl/cystinyl aminopeptidase	-1.678
FGF5: fibroblast growth factor 5	-1.678
UROS: uroporphyrinogen III synthase	-1.679
GMNN: geminin, DNA replication inhibitor	-1.679
KLHL21: kelch-like family member 21	-1.68
MARCH3: membrane-associated ring finger (C3HC4) 3, E3 ubiquitin protein ligase	-1.682
C10orf54: chromosome 10 open reading frame 54	-1.684
CSRNP3: cysteine-serine-rich nuclear protein 3	-1.684
PPARG: peroxisome proliferator-activated receptor gamma	-1.685
NAV2: neuron navigator 2	-1.686
EDNRB: endothelin receptor type B	-1.688
LMO3: LIM domain only 3 (rhombotin-like 2)	-1.688
GNA14: guanine nucleotide binding protein (G protein), alpha 14	-1.689
FAM20B: family with sequence similarity 20, member B	-1.692
BCR: breakpoint cluster region	-1.696
NIPSNAP1: nipsnap homolog 1 (C. elegans)	-1.696
SLC7A8: solute carrier family 7 (amino acid transporter light chain, L system), member 8	-1.696
CROT: carnitine O-octanoyltransferase	-1.699
UBE2N: ubiquitin-conjugating enzyme E2N	-1.704
GJA1: gap junction protein, alpha 1, 43kDa	-1.707
PEG10: paternally expressed 10	-1.707
PDE3A: phosphodiesterase 3A, cGMP-inhibited	-1.707
DOCK4: dedicator of cytokinesis 4	-1.709
MYADM: myeloid-associated differentiation marker	-1.71
CCND3: cyclin D3	-1.711
P4HA3: prolyl 4-hydroxylase, alpha polypeptide III	-1.715
TGOLN2: trans-golgi network protein 2	-1.715
HAX1: HCLS1 associated protein X-1	-1.715

PEF1: penta-EF-hand domain containing 1	-1.715
BMPER: BMP binding endothelial regulator	-1.718
KCTD16: potassium channel tetramerization domain containing 16	-1.719
INTS1: integrator complex subunit 1	-1.724
SBDS: Shwachman-Bodian-Diamond syndrome	-1.725
NRG1: neuregulin 1	-1.726
CAPN5: calpain 5	-1.727
ABHD14B: abhydrolase domain containing 14B	-1.728
ADK: adenosine kinase	-1.731
PTGS2: prostaglandin-endoperoxide synthase 2 (prostaglandin G/H synthase and cyclooxygenase)	-1.733
FAM107B: family with sequence similarity 107, member B	-1.734
NR1D2: nuclear receptor subfamily 1, group D, member 2	-1.737
GSTCD: glutathione S-transferase, C-terminal domain containing	-1.738
B4GALNT1: beta-1,4-N-acetyl-galactosaminyl transferase 1	-1.742
MMP16: matrix metallopeptidase 16 (membrane-inserted)	-1.745
WNK1: WNK lysine deficient protein kinase 1	-1.75
LCA5: Leber congenital amaurosis 5	-1.751
THOC5: THO complex 5	-1.753
B3GNT5: UDP-GlcNAc:betaGal beta-1,3-N-acetylglucosaminyltransferase 5	-1.755
ADAMTS6: ADAM metallopeptidase with thrombospondin type 1 motif, 6	-1.756
GNPDA1: glucosamine-6-phosphate deaminase 1	-1.758
SYNGR2: synaptogyrin 2	-1.758
MARCH8: membrane-associated ring finger (C3HC4) 8, E3 ubiquitin protein ligase	-1.759
CERS2: ceramide synthase 2	-1.761
SCARA5: scavenger receptor class A, member 5 (putative)	-1.762
LRCH2: leucine-rich repeats and calponin homology (CH) domain containing 2	-1.768
EOGT: EGF domain-specific O-linked N-acetylglucosamine (GlcNAc) transferase	-1.77
PRKACA: protein kinase, cAMP-dependent, catalytic, alpha	-1.772
CST1: cystatin SN	-1.773
KCND1: potassium voltage-gated channel, Shal-related subfamily, member 1	-1.777
SCN2A: sodium channel, voltage-gated, type II, alpha subunit	-1.778
SLC27A4: solute carrier family 27 (fatty acid transporter), member 4	-1.781
CELF1: CUGBP, Elav-like family member 1	-1.789
TMC7: transmembrane channel-like 7	-1.79
PTGES: prostaglandin E synthase	-1.794
HOOK3: hook homolog 3 (Drosophila)	-1.796
RWDD4: RWD domain containing 4	-1.798
ESM1: endothelial cell-specific molecule 1	-1.803
TMED1: transmembrane emp24 protein transport domain containing 1	-1.807
UCP2: uncoupling protein 2 (mitochondrial, proton carrier)	-1.808
C2orf61: chromosome 2 open reading frame 61	-1.811
MAP3K2: mitogen-activated protein kinase kinase kinase 2	-1.812

RAD21: RAD21 homolog (S. pombe)	-1.814
SYTL5: synaptotagmin-like 5	-1.819
RNF13: ring finger protein 13	-1.823
GHR: growth hormone receptor	-1.825
RDH11: retinol dehydrogenase 11 (all-trans/9-cis/11-cis)	-1.826
LGR5: leucine-rich repeat containing G protein-coupled receptor 5	-1.826
BTRC: beta-transducin repeat containing E3 ubiquitin protein ligase	-1.828
CTDSP1: CTD (carboxy-terminal domain, RNA polymerase II, polypeptide A) small phosphatase 1	-1.829
ARRDC2: arrestin domain containing 2	-1.83
TOPORS: topoisomerase I binding, arginine/serine-rich, E3 ubiquitin protein ligase	-1.831
SLC2A13: solute carrier family 2 (facilitated glucose transporter), member 13	-1.831
ANO3: anoctamin 3	-1.84
RAPGEF6: Rap guanine nucleotide exchange factor (GEF) 6	-1.841
NDST1: N-deacetylase/N-sulfotransferase (heparan glucosaminyl) 1	-1.842
LIMD1-AS1: LIMD1 antisense RNA 1	-1.845
NDRG1: N-myc downstream regulated 1	-1.851
HSD17B11: hydroxysteroid (17-beta) dehydrogenase 11	-1.858
IFNAR1: interferon (alpha, beta and omega) receptor 1	-1.862
KY: kyphoscoliosis peptidase	-1.863
VSNL1: visinin-like 1	-1.864
PCDH10: protocadherin 10	-1.87
PCDH9: protocadherin 9	-1.871
ARPP21: cAMP-regulated phosphoprotein, 21kDa	-1.876
FAM89B: family with sequence similarity 89, member B	-1.88
PIKFYVE: phosphoinositide kinase, FYVE finger containing	-1.881
TNXB: tenascin XB	-1.885
RNASEL: ribonuclease L (2',5'-oligoisoadenylate synthetase-dependent)	-1.887
KLHL41: kelch-like family member 41	-1.894
AP1S3: adaptor-related protein complex 1, sigma 3 subunit	-1.903
SESN3: sestrin 3	-1.904
GALNT1: UDP-N-acetyl-alpha-D-galactosamine:polypeptide N-acetylgalactosaminyltransferase 1 (GalNAc-T1)	-1.908
GAP43: growth associated protein 43	-1.909
DGCR2: DiGeorge syndrome critical region gene 2	-1.914
SLC14A1: solute carrier family 14 (urea transporter), member 1 (Kidd blood group)	-1.921
AAGAB: alpha- and gamma-adaptin binding protein	-1.924
CIT: citron (rho-interacting, serine/threonine kinase 21)	-1.924
C12orf56: chromosome 12 open reading frame 56	-1.933
BMP6: bone morphogenetic protein 6	-1.933
SCGB3A2: secretoglobin, family 3A, member 2	-1.938
PRSS3P2: protease, serine, 3 pseudogene 2	-1.945
HAS1: hyaluronan synthase 1	-1.962
UPK1B: uroplakin 1B	-1.964

LOC344967: acyl-CoA thioesterase 7 pseudogene	-1.966
GDF10: growth differentiation factor 10	-1.967
TBC1D8: TBC1 domain family, member 8 (with GRAM domain)	-1.969
TAF12: TAF12 RNA polymerase II, TATA box binding protein (TBP)-associated factor, 20kDa	-1.969
LYPD1: LY6/PLAUR domain containing 1	-1.97
STK38: serine/threonine kinase 38	-1.977
MALL: mal, T-cell differentiation protein-like	-1.98
PLOD3: procollagen-lysine, 2-oxoglutarate 5-dioxygenase 3	-1.984
SCN9A: sodium channel, voltage-gated, type IX, alpha subunit	-1.986
SLC1A1: solute carrier family 1 (neuronal/epithelial high affinity glutamate transporter, system Xag), member 1	-1.987
LYVE1: lymphatic vessel endothelial hyaluronan receptor 1	-1.99
MYO9A: myosin IXA	-1.996
NR4A2: nuclear receptor subfamily 4, group A, member 2	-2
FYCO1: FYVE and coiled-coil domain containing 1	-2
PPTC7: PTC7 protein phosphatase homolog (S. cerevisiae)	-2.017
PC: pyruvate carboxylase	-2.03
VAMP3: vesicle-associated membrane protein 3	-2.031
LPGAT1: lysophosphatidylglycerol acyltransferase 1	-2.037
TMEM245: transmembrane protein 245	-2.045
EDEM3: ER degradation enhancer, mannosidase alpha-like 3	-2.046
ENTPD4: ectonucleoside triphosphate diphosphohydrolase 4	-2.066
ABCD3: ATP-binding cassette, sub-family D (ALD), member 3	-2.069
PTHLH: parathyroid hormone-like hormone	-2.073
SLC39A10: solute carrier family 39 (zinc transporter), member 10	-2.074
MFAP3: microfibrillar-associated protein 3	-2.088
HTR4: 5-hydroxytryptamine (serotonin) receptor 4, G protein-coupled	-2.09
PODXL: podocalyxin-like	-2.097
GPR1: G protein-coupled receptor 1	-2.097
ST3GAL1: ST3 beta-galactoside alpha-2,3-sialyltransferase 1	-2.12
GRAMD1C: GRAM domain containing 1C	-2.123
NR4A1: nuclear receptor subfamily 4, group A, member 1	-2.126
FABP3: fatty acid binding protein 3, muscle and heart (mammary-derived growth inhibitor)	-2.127
AREG/AREGB: amphiregulin	-2.139
TAPT1: transmembrane anterior posterior transformation 1	-2.15
SEMA3A: sema domain, immunoglobulin domain (Ig), short basic domain, secreted, (semaphorin) 3A	-2.153
IFNE: interferon, epsilon	-2.157
GRIK1: glutamate receptor, ionotropic, kainate 1	-2.17
S100A16: S100 calcium binding protein A16	-2.173
AKR1B10: aldo-keto reductase family 1, member B10 (aldose reductase)	-2.201
RPN1: ribophorin I	-2.218
PAIP1: poly(A) binding protein interacting protein 1	-2.22
KLHL13: kelch-like family member 13	-2.233

PLEKHB2: pleckstrin homology domain containing, family B (evectins) member 2	-2.234
PHF19: PHD finger protein 19	-2.246
GABRB1: gamma-aminobutyric acid (GABA) A receptor, beta 1	-2.247
PTGIS: prostaglandin I2 (prostacyclin) synthase	-2.249
CYFIP2: cytoplasmic FMR1 interacting protein 2	-2.264
PAQR5: progestin and adipoQ receptor family member V	-2.27
FGL2: fibrinogen-like 2	-2.325
TRPC6: transient receptor potential cation channel, subfamily C, member 6	-2.332
PGM2L1: phosphoglucomutase 2-like 1	-2.355
PLXNA2: plexin A2	-2.374
ITPR3: inositol 1,4,5-trisphosphate receptor, type 3	-2.381
B3GNT2: UDP-GlcNAc:betaGal beta-1,3-N-acetylglucosaminyltransferase 2	-2.384
TNFSF15: tumor necrosis factor (ligand) superfamily, member 15	-2.393
LINC00161: long intergenic non-protein coding RNA 161	-2.414
HAS2: hyaluronan synthase 2	-2.426
HHIP: hedgehog interacting protein	-2.427
CTDSPL: CTD (carboxy-terminal domain, RNA polymerase II, polypeptide A) small phosphatase-like	-2.449
LPHN3: latrophilin 3	-2.488
AHNAK2: AHNAK nucleoprotein 2	-2.538
HMMR: hyaluronan-mediated motility receptor (RHAMM)	-2.559
IGFBP3: insulin-like growth factor binding protein 3	-2.583
CDON: cell adhesion associated, oncogene regulated	-2.603
KLF4: Kruppel-like factor 4 (gut)	-2.618
FAM214B: family with sequence similarity 214, member B	-2.643
BIRC3: baculoviral IAP repeat containing 3	-2.668
PPP2R3A: protein phosphatase 2, regulatory subunit B'', alpha	-2.683
ITGA6: integrin, alpha 6	-2.707
FAM167A: family with sequence similarity 167, member A	-2.777
ADCY6: adenylate cyclase 6	-2.816
DHRS9: dehydrogenase/reductase (SDR family) member 9	-2.818
NAGPA: N-acetylglucosamine-1-phosphodiester alpha-N-acetylglucosaminidase	-2.965
YES1: v-yes-1 Yamaguchi sarcoma viral oncogene homolog 1	-3.043
RECK: reversion-inducing-cysteine-rich protein with kazal motifs	-3.061
NTSR1: neurotensin receptor 1 (high affinity)	-3.129
TEK: TEK tyrosine kinase, endothelial	-3.188
CASP4: caspase 4, apoptosis-related cysteine peptidase	-3.294
SERPINB2: serpin peptidase inhibitor, clade B (ovalbumin), member 2	-3.447
DEPDC1: DEP domain containing 1	-3.521
TMEFF2: transmembrane protein with EGF-like and two follistatin-like domains 2	-3.524
FAM73A: family with sequence similarity 73, member A	-3.538
ADAMTS4: ADAM metallopeptidase with thrombospondin type 1 motif, 4	-3.59
AMIGO2: adhesion molecule with Ig-like domain 2	-3.689

RARG: retinoic acid receptor, gamma	-3.779
RRAS: related RAS viral (r-ras) oncogene homolog	-3.816
SLC39A1: solute carrier family 39 (zinc transporter), member 1	-3.832
THBD: thrombomodulin	-4.256
ROCK1: Rho-associated, coiled-coil containing protein kinase 1	-11.787

Table C. Genes whose expression is altered by ZIPK knockdown.

Gene Symbol: Gene Name	Fold Change
SCML2: sex comb on midleg-like 2 (Drosophila)	3.174
FAM49B: family with sequence similarity 49, member B	2.711
HDAC1: histone deacetylase 1	2.613
FRK: fyn-related kinase	2.373
DDAH1: dimethylarginine dimethylaminohydrolase 1	2.339
KIAA1467: KIAA1467	2.221
CDC25A: cell division cycle 25A	2.212
DCP2: decapping mRNA 2	2.183
ZNF483: zinc finger protein 483	2.07
EIF5A2: eukaryotic translation initiation factor 5A2	2.044
SLC35G1: solute carrier family 35, member G1	2.008
CCNE1: cyclin E1	1.995
MBOAT1: membrane bound O-acyltransferase domain containing 1	1.992
MAP7D2: MAP7 domain containing 2	1.978
ZNF138: zinc finger protein 138	1.972
MGC57346: uncharacterized LOC401884	1.971
SERPINB9: serpin peptidase inhibitor, clade B (ovalbumin), member 9	1.942
ZNF678: zinc finger protein 678	1.94
SNORD25: small nucleolar RNA, C/D box 25	1.932
ZNF43: zinc finger protein 43	1.931
NAP1L3: nucleosome assembly protein 1-like 3	1.91
ZC3HAV1L: zinc finger CCCH-type, antiviral 1-like	1.907
ZNF100: zinc finger protein 100	1.907
MCM10: minichromosome maintenance complex component 10	1.892
SPIN4: spindlin family, member 4	1.886
MICB: MHC class I polypeptide-related sequence B	1.866
ADAM21: ADAM metallopeptidase domain 21	1.865
E2F8: E2F transcription factor 8	1.857
CLSPN: claspin	1.852
CELF2: CUGBP, Elav-like family member 2	1.838
C8orf44-SGK3/SGK3: serum/glucocorticoid regulated kinase family, member 3	1.834
FAM60A: family with sequence similarity 60, member A	1.833
UBE2D1: ubiquitin-conjugating enzyme E2D 1	1.83
CDK6: cyclin-dependent kinase 6	1.819
DHRS11: dehydrogenase/reductase (SDR family) member 11	1.814
MTF2: metal response element binding transcription factor 2	1.812
ZNF680: zinc finger protein 680	1.811
GINS1: GINS complex subunit 1 (Psf1 homolog)	1.807
PRIM1: primase, DNA, polypeptide 1 (49kDa)	1.804

CPM: carboxypeptidase M	1.801
GXYLT1: glucoside xylosyltransferase 1	1.8
ZNF93: zinc finger protein 93	1.796
ATAD5: ATPase family, AAA domain containing 5	1.765
C10orf12: chromosome 10 open reading frame 12	1.764
ZNF253: zinc finger protein 253	1.753
CCNE2: cyclin E2	1.748
HPGD: hydroxyprostaglandin dehydrogenase 15-(NAD)	1.746
STIL: SCL/TAL1 interrupting locus	1.732
SLC16A14: solute carrier family 16, member 14 (monocarboxylic acid transporter 14)	1.731
KPNA5: karyopherin alpha 5 (importin alpha 6)	1.723
AGK: acylglycerol kinase	1.722
LCOR: ligand dependent nuclear receptor corepressor	1.716
FBXO27: F-box protein 27	1.714
ZNF708: zinc finger protein 708	1.71
HIST1H2BJ/HIST1H2BK: histone cluster 1, H2bk	1.694
RAD51AP1: RAD51 associated protein 1	1.68
FNBP1L: formin binding protein 1-like	1.68
BPNT1: 3'(2'), 5'-bisphosphate nucleotidase 1	1.674
ZNF107: zinc finger protein 107	1.668
CDC6: cell division cycle 6	1.667
PRTG: protogenin	1.665
POLR3G: polymerase (RNA) III (DNA directed) polypeptide G (32kD)	1.664
ESCO2: establishment of sister chromatid cohesion N-acetyltransferase 2	1.663
GINS2: GINS complex subunit 2 (Psf2 homolog)	1.659
TP53INP1: tumor protein p53 inducible nuclear protein 1	1.655
CARS2: cysteinyl-tRNA synthetase 2, mitochondrial (putative)	1.652
YOD1: YOD1 deubiquitinase	1.649
TIA1: TIA1 cytotoxic granule-associated RNA binding protein	1.647
ZNF92: zinc finger protein 92	1.645
SCML1: sex comb on midleg-like 1 (Drosophila)	1.641
SASS6: spindle assembly 6 homolog (C. elegans)	1.639
FAM111B: family with sequence similarity 111, member B	1.633
MCM7: minichromosome maintenance complex component 7	1.633
DNA2: DNA replication helicase/nuclease 2	1.633
DEPDC1B: DEP domain containing 1B	1.633
C11orf82: chromosome 11 open reading frame 82	1.627
MLLT4: myeloid/lymphoid or mixed-lineage leukemia (trithorax homolog, Drosophila); translocated to, 4	1.627
SNORD30: small nucleolar RNA, C/D box 30	1.625
ANKRD46: ankyrin repeat domain 46	1.624
KLHDC3: kelch domain containing 3	1.622
DNAJC27: DnaJ (Hsp40) homolog, subfamily C, member 27	1.62

GCA: grancalcin, EF-hand calcium binding protein	1.62
ZNF141: zinc finger protein 141	1.615
ASF1B: anti-silencing function 1B histone chaperone	1.615
TMEM170A: transmembrane protein 170A	1.613
CCDC125: coiled-coil domain containing 125	1.609
MCM6: minichromosome maintenance complex component 6	1.607
DCBLD1: discoidin, CUB and LCCL domain containing 1	1.604
SNORD14E: small nucleolar RNA, C/D box 14E	1.603
GPAM: glycerol-3-phosphate acyltransferase, mitochondrial	1.601
ATG2B: autophagy related 2B	1.6
CLDN1: claudin 1	1.599
ATP6V1D: ATPase, H+ transporting, lysosomal 34kDa, V1 subunit D	1.599
RNF144B: ring finger protein 144B	1.596
TMEM144: transmembrane protein 144	1.596
SPTLC2: serine palmitoyltransferase, long chain base subunit 2	1.596
ZNF35: zinc finger protein 35	1.592
ZNF273: zinc finger protein 273	1.584
MARS2: methionyl-tRNA synthetase 2, mitochondrial	1.583
ZNF91: zinc finger protein 91	1.583
C9orf72: chromosome 9 open reading frame 72	1.578
MTBP: Mdm2, transformed 3T3 cell double minute 2, p53 binding protein (mouse) binding protein, 104kDa	1.577
ZNF738: zinc finger protein 738	1.576
DTL: denticleless E3 ubiquitin protein ligase homolog (Drosophila)	1.57
EXOC6: exocyst complex component 6	1.57
ZNF682: zinc finger protein 682	1.567
ZNF681: zinc finger protein 681	1.565
CEP97: centrosomal protein 97kDa	1.564
VWA9: von Willebrand factor A domain containing 9	1.564
LRRC1: leucine rich repeat containing 1	1.561
SMPD4: sphingomyelin phosphodiesterase 4, neutral membrane (neutral sphingomyelinase-3)	1.56
GNL3L: guanine nucleotide binding protein-like 3 (nucleolar)-like	1.559
RBL1: retinoblastoma-like 1 (p107)	1.559
CCP110: centriolar coiled coil protein 110kDa	1.558
PARP16: poly (ADP-ribose) polymerase family, member 16	1.556
POLQ: polymerase (DNA directed), theta	1.554
RAB3IP: RAB3A interacting protein	1.553
MCM5: minichromosome maintenance complex component 5	1.553
NCAM2: neural cell adhesion molecule 2	1.551
ENTPD7: ectonucleoside triphosphate diphosphohydrolase 7	1.55
ZNF254: zinc finger protein 254	1.548
EXO1: exonuclease 1	1.548

MCM3: minichromosome maintenance complex component 3	1.546
H2AFY2: H2A histone family, member Y2	1.545
EML4: echinoderm microtubule associated protein like 4	1.545
HMGB2: high mobility group box 2	1.545
APAF1: apoptotic peptidase activating factor 1	1.542
AMER1: APC membrane recruitment protein 1	1.542
CTPS2: CTP synthase 2	1.541
HSPA4L: heat shock 70kDa protein 4-like	1.541
MSH2: mutS homolog 2, colon cancer, nonpolyposis type 1 (E. coli)	1.539
ATP11A: ATPase, class VI, type 11A	1.539
SGOL1: shugoshin-like 1 (S. pombe)	1.538
ZNF431: zinc finger protein 431	1.537
STRBP: spermatid perinuclear RNA binding protein	1.535
SYPL1: synaptophysin-like 1	1.531
CEP85: centrosomal protein 85kDa	1.531
VPS36: vacuolar protein sorting 36 homolog (S. cerevisiae)	1.528
SKP2: S-phase kinase-associated protein 2, E3 ubiquitin protein ligase	1.527
PDHA1: pyruvate dehydrogenase (lipoamide) alpha 1	1.523
NKIRAS2: NFKB inhibitor interacting Ras-like 2	1.521
HIST1H2AB/HIST1H2AE: histone cluster 1, H2ae	1.521
CWF19L1: CWF19-like 1, cell cycle control (S. pombe)	1.521
PCTP: phosphatidylcholine transfer protein	1.519
E2F5: E2F transcription factor 5, p130-binding	1.519
ZNF713: zinc finger protein 713	1.517
TTF2: transcription termination factor, RNA polymerase II	1.516
ATAD2: ATPase family, AAA domain containing 2	1.516
CHAF1B: chromatin assembly factor 1, subunit B (p60)	1.516
ZNF826P: zinc finger protein 826, pseudogene	1.515
TIPIN: TIMELESS interacting protein	1.515
ZFYVE16: zinc finger, FYVE domain containing 16	1.514
FDXACB1: ferredoxin-fold anticodon binding domain containing 1	1.513
PLK4: polo-like kinase 4	1.512
SNORA27: small nucleolar RNA, H/ACA box 27	1.511
NUP54: nucleoporin 54kDa	1.511
RTKN2: rhotekin 2	1.511
WEE1: WEE1 homolog (S. pombe)	1.51
CHD7: chromodomain helicase DNA binding protein 7	1.509
DSCC1: DNA replication and sister chromatid cohesion 1	1.507
RAD51: RAD51 recombinase	1.506
SNORD78: small nucleolar RNA, C/D box 78	1.505
KIAA1147: KIAA1147	1.505
ACER2: alkaline ceramidase 2	1.505

EPHA5: EPH receptor A5	1.505
CEP57: centrosomal protein 57kDa	1.503
BUB1B: BUB1 mitotic checkpoint serine/threonine kinase B	1.502
PSTPIP2: proline-serine-threonine phosphatase interacting protein 2	1.502
SNORD22: small nucleolar RNA, C/D box 22	1.502
N4BP2: NEDD4 binding protein 2	1.501
RPIA: ribose 5-phosphate isomerase A	1.5
KRT7: keratin 7	-1.504
SEL1L3: sel-1 suppressor of lin-12-like 3 (C. elegans)	-1.504
ETS2: v-ets erythroblastosis virus E26 oncogene homolog 2 (avian)	-1.505
MSI2: musashi RNA-binding protein 2	-1.506
ARHGAP6: Rho GTPase activating protein 6	-1.508
CREB3L1: cAMP responsive element binding protein 3-like 1	-1.509
FAM214B: family with sequence similarity 214, member B	-1.51
TLR4: toll-like receptor 4	-1.511
CCDC103: coiled-coil domain containing 103	-1.511
PODNL1: podocan-like 1	-1.512
LIN7A: lin-7 homolog A (C. elegans)	-1.514
GK3P: glycerol kinase 3 pseudogene	-1.514
CHST11: carbohydrate (chondroitin 4) sulfotransferase 11	-1.514
ARMCX2: armadillo repeat containing, X-linked 2	-1.516
F2RL2: coagulation factor II (thrombin) receptor-like 2	-1.518
NR1D2: nuclear receptor subfamily 1, group D, member 2	-1.518
VASN: vasorin	-1.521
CCND2: cyclin D2	-1.521
FTSJ1: FtsJ RNA methyltransferase homolog 1 (E. coli)	-1.523
TPCN1: two pore segment channel 1	-1.524
PID1: phosphotyrosine interaction domain containing 1	-1.524
TMEM119: transmembrane protein 119	-1.524
CCDC110: coiled-coil domain containing 110	-1.525
DUSP5: dual specificity phosphatase 5	-1.525
IFI30: interferon, gamma-inducible protein 30	-1.526
CCS: copper chaperone for superoxide dismutase	-1.527
NAA25: N(alpha)-acetyltransferase 25, NatB auxiliary subunit	-1.528
G0S2: G0/G1switch 2	-1.529
LOXL1: lysyl oxidase-like 1	-1.53
C9orf47: chromosome 9 open reading frame 47	-1.532
FAM87B: family with sequence similarity 87, member B	-1.533
ANGPTL2: angiopoietin-like 2	-1.533
TUBA4A: tubulin, alpha 4a	-1.534
MAPK6: mitogen-activated protein kinase 6	-1.534
TSLP: thymic stromal lymphopoietin	-1.537

STARD5: StAR-related lipid transfer (START) domain containing 5	-1.54
TCP11L2: t-complex 11, testis-specific-like 2	-1.541
TGFB3: transforming growth factor, beta 3	-1.543
SAT1: spermidine/spermine N1-acetyltransferase 1	-1.544
DUSP4: dual specificity phosphatase 4	-1.545
PREP: prolyl endopeptidase	-1.545
NFASC: neurofascin	-1.545
EMR2: egf-like module containing, mucin-like, hormone receptor-like 2	-1.545
NOTCH3: notch 3	-1.55
PGF: placental growth factor	-1.55
CRISPLD2: cysteine-rich secretory protein LCCL domain containing 2	-1.551
HECW2: HECT, C2 and WW domain containing E3 ubiquitin protein ligase 2	-1.555
PON2: paraoxonase 2	-1.555
PEAR1: platelet endothelial aggregation receptor 1	-1.556
AGTR1: angiotensin II receptor, type 1	-1.556
ZNF395: zinc finger protein 395	-1.556
SATB1: SATB homeobox 1	-1.558
SHROOM3: shroom family member 3	-1.559
MYOCD: myocardin	-1.561
PRELP: proline/arginine-rich end leucine-rich repeat protein	-1.564
SPTLC3: serine palmitoyltransferase, long chain base subunit 3	-1.565
PI15: peptidase inhibitor 15	-1.565
NDRG1: N-myc downstream regulated 1	-1.565
SLC7A7: solute carrier family 7 (amino acid transporter light chain, y+L system), member 7	-1.565
NFAT5: nuclear factor of activated T-cells 5, tonicity-responsive	-1.565
WDR1: WD repeat domain 1	-1.566
COMMD8: COMM domain containing 8	-1.567
GDE1: glycerophosphodiester phosphodiesterase 1	-1.567
GSTT2/GSTT2B: glutathione S-transferase theta 2	-1.568
ADA: adenosine deaminase	-1.57
BOC: BOC cell adhesion associated, oncogene regulated	-1.572
PGM5: phosphoglucomutase 5	-1.576
FZD7: frizzled family receptor 7	-1.578
MALL: mal, T-cell differentiation protein-like	-1.583
TOR1AIP2: torsin A interacting protein 2	-1.584
CCDC91: coiled-coil domain containing 91	-1.592
NUPR1: nuclear protein, transcriptional regulator, 1	-1.592
C1orf110: chromosome 1 open reading frame 110	-1.592
FXYD6: FXYD domain containing ion transport regulator 6	-1.594
CD82: CD82 molecule	-1.597
TNPO3: transportin 3	-1.597
TNFRSF21: tumor necrosis factor receptor superfamily, member 21	-1.599

IMMT: inner membrane protein, mitochondrial	-1.599
HHIP: hedgehog interacting protein	-1.6
CNEP1R1: CTD nuclear envelope phosphatase 1 regulatory subunit 1	-1.6
RAB5B: RAB5B, member RAS oncogene family	-1.6
SERPIND1: serpin peptidase inhibitor, clade D (heparin cofactor), member 1	-1.603
THBD: thrombomodulin	-1.611
RIN2: Ras and Rab interactor 2	-1.611
EVI2B: ecotropic viral integration site 2B	-1.612
TRPV4: transient receptor potential cation channel, subfamily V, member 4	-1.615
LOXL4: lysyl oxidase-like 4	-1.615
GPR4: G protein-coupled receptor 4	-1.617
TPPP3: tubulin polymerization-promoting protein family member 3	-1.619
GPR1: G protein-coupled receptor 1	-1.619
CDK18: cyclin-dependent kinase 18	-1.622
ATP13A2: ATPase type 13A2	-1.625
RNF141: ring finger protein 141	-1.628
SLC16A4: solute carrier family 16, member 4 (monocarboxylic acid transporter 5)	-1.629
LRP12: low density lipoprotein receptor-related protein 12	-1.63
TLE3: transducin-like enhancer of split 3 (E(sp1) homolog, Drosophila)	-1.63
GPRC5A: G protein-coupled receptor, family C, group 5, member A	-1.631
SLC6A6: solute carrier family 6 (neurotransmitter transporter, taurine), member 6	-1.631
MYOZ2: myozenin 2	-1.633
TOB1: transducer of ERBB2, 1	-1.635
HIPK2: homeodomain interacting protein kinase 2	-1.636
MAPK8: mitogen-activated protein kinase 8	-1.64
PLD1: phospholipase D1, phosphatidylcholine-specific	-1.645
CPA4: carboxypeptidase A4	-1.653
PDE4DIP: phosphodiesterase 4D interacting protein	-1.656
SERTAD4: SERTA domain containing 4	-1.657
GJA1: gap junction protein, alpha 1, 43kDa	-1.663
LPCAT2: lysophosphatidylcholine acyltransferase 2	-1.664
JAG1: jagged 1	-1.664
TNXB: tenascin XB	-1.666
PDE7A: phosphodiesterase 7A	-1.669
MTMR6: myotubularin related protein 6	-1.67
CD22: CD22 molecule	-1.672
CYGB: cytoglobin	-1.673
AHNAK2: AHNAK nucleoprotein 2	-1.677
APOLD1: apolipoprotein L domain containing 1	-1.685
NCOA6: nuclear receptor coactivator 6	-1.687
RNF13: ring finger protein 13	-1.688
C10orf54: chromosome 10 open reading frame 54	-1.688

RDH5: retinol dehydrogenase 5 (11-cis/9-cis)	-1.688
TTYH3: tweety homolog 3 (Drosophila)	-1.689
SIK3: SIK family kinase 3	-1.7
CDC42BPA: CDC42 binding protein kinase alpha (DMPK-like)	-1.7
CEP19: centrosomal protein 19kDa	-1.702
ASS1: argininosuccinate synthase 1	-1.705
SMG7: SMG7 nonsense mediated mRNA decay factor	-1.706
INA: internexin neuronal intermediate filament protein, alpha	-1.707
SLC2A12: solute carrier family 2 (facilitated glucose transporter), member 12	-1.709
HBEGF: heparin-binding EGF-like growth factor	-1.711
CNN1: calponin 1, basic, smooth muscle	-1.716
AREG/AREGB: amphiregulin	-1.72
MASP1: mannan-binding lectin serine peptidase 1 (C4/C2 activating component of Ra-reactive factor)	-1.722
ST3GAL1: ST3 beta-galactoside alpha-2,3-sialyltransferase 1	-1.722
DENND1B: DENN/MADD domain containing 1B	-1.724
SYTL3: synaptotagmin-like 3	-1.725
SYNC: syncoilin, intermediate filament protein	-1.728
PER3: period circadian clock 3	-1.731
MYO10: myosin X	-1.732
SLC6A9: solute carrier family 6 (neurotransmitter transporter, glycine), member 9	-1.733
KMT2D: lysine (K)-specific methyltransferase 2D	-1.737
SLC19A3: solute carrier family 19, member 3	-1.737
TCF7: transcription factor 7 (T-cell specific, HMG-box)	-1.744
DAB2: Dab, mitogen-responsive phosphoprotein, homolog 2 (Drosophila)	-1.747
VTI1B: vesicle transport through interaction with t-SNAREs 1B	-1.756
ERRFI1: ERBB receptor feedback inhibitor 1	-1.757
VCAM1: vascular cell adhesion molecule 1	-1.76
KCNK3: potassium channel, subfamily K, member 3	-1.774
PSME4: proteasome (prosome, macropain) activator subunit 4	-1.777
TNFSF15: tumor necrosis factor (ligand) superfamily, member 15	-1.78
MAML3: mastermind-like 3 (Drosophila)	-1.786
CD248: CD248 molecule, endosialin	-1.79
ISM1: isthmin 1, angiogenesis inhibitor	-1.793
FABP3: fatty acid binding protein 3, muscle and heart (mammary-derived growth inhibitor)	-1.799
KCND1: potassium voltage-gated channel, Shal-related subfamily, member 1	-1.799
FAF2: Fas associated factor family member 2	-1.811
ITGA10: integrin, alpha 10	-1.826
TNFAIP2: tumor necrosis factor, alpha-induced protein 2	-1.829
FSTL3: follistatin-like 3 (secreted glycoprotein)	-1.829
R3HDM2: R3H domain containing 2	-1.832
PDK4: pyruvate dehydrogenase kinase, isozyme 4	-1.833

RTN4RL1: reticulon 4 receptor-like 1	-1.846
FLVCR2: feline leukemia virus subgroup C cellular receptor family, member 2	-1.852
CRYAB: crystallin, alpha B	-1.859
FCGR1A: Fc fragment of IgG, high affinity Ia, receptor (CD64)	-1.863
BMP2K: BMP2 inducible kinase	-1.868
PAQR5: progestin and adipoQ receptor family member V	-1.87
SIRPA: signal-regulatory protein alpha	-1.871
SELENBP1: selenium binding protein 1	-1.874
RAB30: RAB30, member RAS oncogene family	-1.887
LYVE1: lymphatic vessel endothelial hyaluronan receptor 1	-1.887
AGPAT9: 1-acylglycerol-3-phosphate O-acyltransferase 9	-1.887
C16orf45: chromosome 16 open reading frame 45	-1.895
SCGB3A2: secretoglobin, family 3A, member 2	-1.897
AAK1: AP2 associated kinase 1	-1.911
TMEM100: transmembrane protein 100	-1.923
CYTIP: cytohesin 1 interacting protein	-1.93
PLAU: plasminogen activator, urokinase	-1.939
CRIPT: cysteine-rich PDZ-binding protein	-1.943
IL1R1: interleukin 1 receptor, type I	-1.947
PCSK7: proprotein convertase subtilisin/kexin type 7	-1.947
GDPD3: glycerophosphodiester phosphodiesterase domain containing 3	-1.947
FCGR1B: Fc fragment of IgG, high affinity Ib, receptor (CD64)	-1.96
S100A3: S100 calcium binding protein A3	-1.96
GFPT2: glutamine-fructose-6-phosphate transaminase 2	-1.961
CKB: creatine kinase, brain	-1.97
SLC7A8: solute carrier family 7 (amino acid transporter light chain, L system), member 8	-1.972
CCDC68: coiled-coil domain containing 68	-1.974
MED15: mediator complex subunit 15	-1.978
CYB5D2: cytochrome b5 domain containing 2	-2.002
PTGS2: prostaglandin-endoperoxide synthase 2 (prostaglandin G/H synthase and cyclooxygenase)	-2.008
THAP10: THAP domain containing 10	-2.025
TGM2: transglutaminase 2 (C polypeptide, protein-glutamine-gamma-glutamyltransferase)	-2.026
GPX7: glutathione peroxidase 7	-2.035
GPR56: G protein-coupled receptor 56	-2.036
ANGPTL4: angiopoietin-like 4	-2.05
LMOD1: leiomodin 1 (smooth muscle)	-2.108
SH3BGRL3: SH3 domain binding glutamic acid-rich protein like 3	-2.128
CLGN: calmegin	-2.139
CERS2: ceramide synthase 2	-2.146
KLHL41: kelch-like family member 41	-2.146
ADAMTS4: ADAM metallopeptidase with thrombospondin type 1 motif, 4	-2.154

SFT2D2: SFT2 domain containing 2	-2.16
DHRS9: dehydrogenase/reductase (SDR family) member 9	-2.163
MAML2: mastermind-like 2 (Drosophila)	-2.169
COL4A1: collagen, type IV, alpha 1	-2.201
S100A16: S100 calcium binding protein A16	-2.212
ATP1B1: ATPase, Na+/K+ transporting, beta 1 polypeptide	-2.213
CLOCK: clock circadian regulator	-2.234
ANTXR2: anthrax toxin receptor 2	-2.266
LINC00161: long intergenic non-protein coding RNA 161	-2.318
HIP1: huntingtin interacting protein 1	-2.325
FAIM2: Fas apoptotic inhibitory molecule 2	-2.345
GALNT15: UDP-N-acetyl-alpha-D-galactosamine:polypeptide N-acetylgalactosaminyltransferase 15	-2.351
NID1: nidogen 1	-2.596
FAM180A: family with sequence similarity 180, member A	-2.722
DAPK3: death-associated protein kinase 3	-2.725
MED12: mediator complex subunit 12	-3.245
GFRA2: GDNF family receptor alpha 2	-3.289
DPY19L3: dpy-19-like 3 (C. elegans)	-6.745

Table D. Effects of ZIPK and ROCK1 knockdown on cytokine secretion.

Cytokine	Control	ZIPK knockdown	ROCK1 knockdown
IL-1α	0.278 ± 0.061	0.226 ± 0.051	0.432 ± 0.106
IP10 (CXCL10)	0.327 ± 0.042	0.377 ± 0.051	0.367 ± 0.044
I-TAC (CXCL11)	0.311 ± 0.010	0.332 ± 0.012	0.334 ± 0.013
Eotaxin (CCL11)	0.116 ± 0.018	0.130 ± 0.023	0.148 ± 0.021
MCP1 (CCL2)	0.567 ± 0.041	0.405 ± 0.039*	1.014 ± 0.181*
GRO-α (CXCL1)	1.358 ± 0.249	1.028 ± 0.252	1.872 ± 0.145

A Human Custom Multi-Analyte ELISArray kit (CELISA-CMEH0590A) was purchased from Qiagen. The indicated cytokines were assayed in the medium of CASMC transfected with control siRNA (Control) and CASMC transfected with siRNA to ZIPK (ZIPK knockdown) or ROCK1 (ROCK1 knockdown). Positive controls provided with the kit verified the viability of the assay for each cytokine. Negative controls indicated that, of the 6 cytokines listed, IL-1α, MCP1 and GROα were secreted at detectable levels. Values indicate absorbance at 450 nm ± S.E.M. (n = 4). *$p < 0.05$ compared to Control.
